# Purification and functional comparison of nine human Aquaporins produced in *Saccharomyces cerevisiae* for the purpose of biophysical characterization

**DOI:** 10.1038/s41598-017-17095-6

**Published:** 2017-12-04

**Authors:** Frederik Bühring Bjørkskov, Simon Lyngaa Krabbe, Casper Normann Nurup, Julie Winkel Missel, Mariana Spulber, Julie Bomholt, Karen Molbaek, Claus Helix-Nielsen, Kamil Gotfryd, Pontus Gourdon, Per Amstrup Pedersen

**Affiliations:** 1 Department of Biology, Faculty of Science, University of Copenhagen, Universitetsparken 13, 2100 Copenhagen, OE Denmark; 2 Department of Biomedical Sciences, Faculty of Health, University of Copenhagen, Nørre Alle 41, 2200 Copenhagen, N Denmark; 3 Aquaporin A/S, Nymøllevej 78, 2800 Lyngby, Denmark; 4 Department of Environmental Engineering, Technical University of Denmark, 2800 Lyngby, Denmark; 5 Laboratory for Water Biophysics and Membrane Processes, University of Maribor, 2000 Maribor, Slovenia; 6 Department of Experimental Medical Sciences, Lund University, Sölvegatan 19, 2218 Lund, Sweden

## Abstract

The sparse number of high-resolution human membrane protein structures severely restricts our comprehension of molecular physiology and ability to exploit rational drug design. In the search for a standardized, cheap and easily handled human membrane protein production platform, we thoroughly investigated the capacity of *S. cerevisiae* to deliver high yields of prime quality human AQPs, focusing on poorly characterized members including some previously shown to be difficult to isolate. Exploiting GFP labeled forms we comprehensively optimized production and purification procedures resulting in satisfactory yields of all nine AQP targets. We applied the obtained knowledge to successfully upscale purification of histidine tagged human AQP10 produced in large bioreactors. Glycosylation analysis revealed that AQP7 and 12 were O-glycosylated, AQP10 was N-glycosylated while the other AQPs were not glycosylated. We furthermore performed functional characterization and found that AQP 2, 6 and 8 allowed flux of water whereas AQP3, 7, 9, 10, 11 and 12 also facilitated a glycerol flux. In conclusion, our *S. cerevisiae* platform emerges as a powerful tool for isolation of functional, difficult-to-express human membrane proteins suitable for biophysical characterization.

## Introduction

For any living organism integral membrane proteins constitute around 30% of the proteome^1^. This large number reflects that a fundamental requirement for life is the capacity of each living cell to maintain homeostasis; a condition that involves a strictly controlled flow of water and water soluble molecules across the plasma membrane and organelle membranes. Membrane transport can only be understood in detail if we know the high resolution structures of the membrane embedded proteins that mediate the transport. Nevertheless, the number of high resolution structures of integral membrane proteins presently amounts to 712^2^, while currently more than 120,000 protein structures are deposited in the Protein Data Bank^3^. These numbers expose how biased our knowledge is in relation to protein structures. The lack of structural information not only restricts our understanding of cell-biology, but also limits our capacity to implement rational drug design in the search for more target specific compounds, as membrane proteins constitute the most prominent class of drug targets^4,5^.

The great majority of membrane protein structures originates from prokaryotes and only 59 are of human origin^2^. Experimentally resolved prokaryotic protein structures have therefore been used extensively as models for human membrane proteins^6^. In addition to the uncertainties associated with this approach, modelling is limited by the fact that around 85% of all human membrane protein families do not have prokaryotic counterparts^7^. Access to more human membrane protein structures is therefore highly desired.

The road to more high resolution structures of human membrane proteins is, however, paved with severe obstacles related to difficulties in achieving high-level high quality expression, and establishing efficient purification procedures that retain the biological activity of the protein, thereby securing samples suitable for structural biology methods such as X-ray crystallography and cryo-EM. Dealing with these issues is far from being trivial and usually approached by a laborious trial and error strategy.

The ability to control water homeostasis is essential to all cells and achieved through the action of AQP water channels that mediate bidirectional, transmembrane water flow in presence of an osmotic gradient^8^. AQPs therefore play fundamental roles in human physiology^9^ and AQP malfunctioning is associated with a number of human diseases^10^. The selective water permeability of the AQPs has furthermore demonstrated huge potentials in biologically based biomimetic water filtration set ups^11^.

Thirteen different AQPs have been identified in humans^12^. These are traditionally subdivided into three major groups; the orthodox AQPs; AQP0, AQP1, AQP2, AQP4, AQP5, AQP6 and AQP8; the aquaglyceroporins AQP3, AQP7, AQP9 and AQP10 and the super-AQPs, AQP11 and AQP12^13^. High resolution structures have been obtained for four orthodox human AQPs AQP1^14^, AQP2^15^, AQP4^16^ and AQP5^17^ and for AQP0 from cow and sheep^18^. The orthodox AQPs are highly specific for water^12^ except for AQP6 that also transports nitrate^19^ and AQP8 that is permeable to urea^20^. The aquaglyceroporins mediate glycerol flux in addition to water and in some cases other small molecules like urea^21^ and arsenic^22^. A number of small gases like CO_2_^23^ and NH_3_^24^, in addition to H_2_O_2_^25^ and ions^26^ have also been shown to pass through AQPs. Apart from their capacity to mediate a water flux the super-AQPs are less well understood^27^.

The high-resolution crystal structures of orthodox AQPs from mammals have revealed that these are homo-tetramers^14,15,17,28,29^. Each monomer carries a pore made up of six trans membrane α-helices, five connecting loops and two half-helices that penetrate the membrane from opposite sides and assemble into a seventh pseudo transmembrane domain^10^, Supplementary Figure S1. We have previously successfully produced and purified a variety of complex human integral membrane proteins using the *S. cerevisiae* -based PAP1500 platform^30–33^. Notably, we have been able to produce human AQP1 to a membrane protein density of 8%^31^, which is extraordinary high. Here we investigated if our expression system is capable of delivering the high yields required for biophysical, biochemical, structural or pharmacological studies of other human AQPs, too. We focused our study on eight poorly characterized human AQPs 3, 6, 7, 8, 9, 10, 11 and 12, as well as human AQP2 which has previously been recognized as difficult to express^34,35^. A primary structure alignment of the AQPs studied in the present paper is shown in Supplementary Figure S2.The molecular weight of each monomer ranges from 27 kDa to 37 kDa depending on the AQP (http://www.uniprot.org/).We show high yields of most targets as wells as a characterization of their water and glycerol permeabilities.

## Results

### The expression system

**Figure 1:**
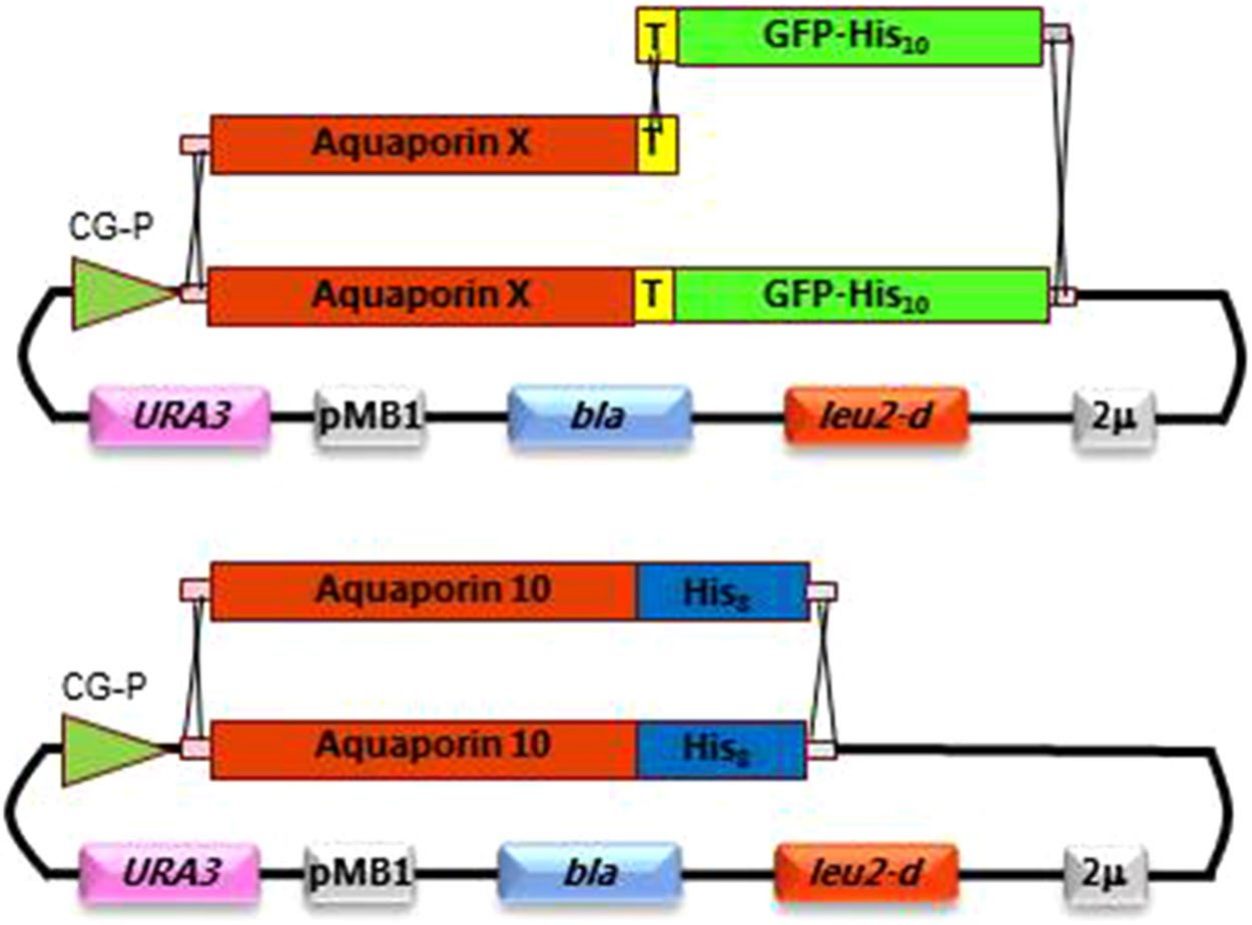
Structural map of the expression plasmids used for production of human AQPs in *S. cerevisiae*. Abbreviations used: CG-P, a hybrid promoter carrying the GAL10 upstream activating sequence fused to the 5′ non-translated leader of the cytochrome-1 gene; AquaporinX, one of the codon optimized human AQPs 2, 3, 6, 7, 8, 9, 10, 11 or 12; T, a Tobacco Etch Virus (TEV) cleavage site; GFP-His_10_, yeast enhanced GFP cDNA fused to ten histidine codons; 2 *μ*, a yeast 2 micron origin of replication; *leu2-d*, a poorly expressed allele of the β-iso-propyl-malate dehydrogenase gene; *bla*, a β-lactamase gene; pMB1, the pMB1origin of replication; *URA3*, the yeast orotidine-5′-phosphate decarboxylase gene. Rapid and efficient construction of all plasmids encoding an AQP-TEV-GFP-His_10_ fusion was carried out by *in vivo* homologous recombination in *S. cerevisiae* between an AQP-TEV PCR fragment, a TEV-GFP-His_10_ PCR fragment and the linearized expression plasmid. The positions of the three required cross-over events are indicated. Construction of the AQP10-His_8_ expression plasmid was generated in a similar way by transforming with an AQP10-His_8_ PCR fragment and the linearized expression plasmid.

We have previously demonstrated that our *S. cerevisiae* platform is able to express human AQP1 to an exceptional high membrane density^31^. We therefore investigated whether high level production of other human AQPs can also be achieved in our PAP1500 expression system^30^. This platform includes a yeast expression plasmid with two important features; i) the plasmid copy number can be increased from 20 to approximately 200 per cell prior to induction of recombinant protein production; ii) expression of the heterologous protein is controlled by a galactose inducible promoter (Fig. 1) and iii) the PAP1500 host strain that concomitantly with recombinant protein production overexpresses the Gal4 transcriptional activator required for galactose induced transcription^36^. Human AQPs 2, 3, 6, 7, 8, 9, 10, 11 and 12 were initially codon optimized for *S. cerevisiae* and produced with a C-terminal TEV-yeGFP-His_10_ tag suitable for identification of optimal conditions for expression and purification. AQP10 was subsequently produced in large scale with only a His_8_ tag, as well.

### AQP accumulation is favored at 15 °C

**Figure 2:**
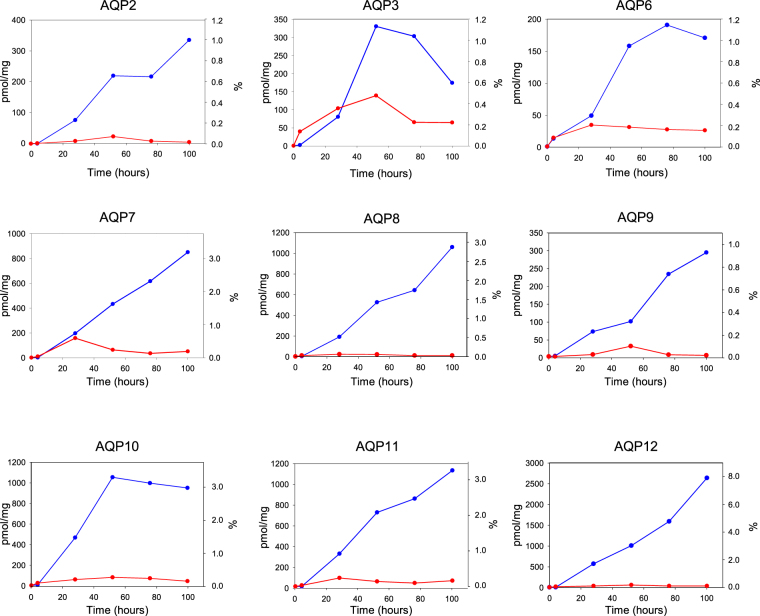
Kinetics of AQP-TEV-GFP-His_10_ accumulation in yeast crude membranes during production at either 15 °C or 30 °C. Yeast strains were grown at room temperature until OD_450_ = 1.0 and cultures were divided in two. One portion was transferred to 15 °C () and the other was transferred to 30 °C (). After thermo-equilibration AQP production was initiated by addition of 2% galactose. Crude membranes were isolated from cells induced for 0, 28, 52, 76 and 100 hours and fluorescence was determined in 25 µg crude membranes and converted to pmol AQP-GFP fusion per mg crude membranes as described in Methods. The pmol/mg was subsequently converted to the percentage of crude membranes constituted by each AQP (%). The molecular weight of GFP was not included in the latter calculation.

Previous experience with our expression platform has shown that membrane proteins accumulate to a significantly higher membrane density if production is performed at 15 °C^31,32^ compared to 30 °C, which is the optimal growth temperature for yeast. To determine if this was also true for the nine AQPs analyzed in the present paper we used the fluorescence from the GFP tag to determine the kinetics of AQP accumulation in yeast membranes at 15 °C and 30 °C. The data in Fig. 2 show that all proteins accumulated to a much higher membrane density at 15 °C compared to 30 °C. In general, at 15 °C each AQP accumulated either linearly with time and did not reach at plateau even after 100 hours of induction or reached a plateau after approximately 76 hours. In contrast at 30 °C accumulation reached a maximum between 24 and 48 hours after induction and subsequently leveled off again. The maximal amount of accumulated GFP fluorescence was between 2.4 and 44 times lower at 30 °C compared to 15 °C. Figure 2 also reveals that based on GFP fluorescence the AQPs constituted between 1% and 8% of the crude membrane protein.

### AQPs accumulate as full length proteins in the *S. cerevisiae* membranes

**Figure 3:**
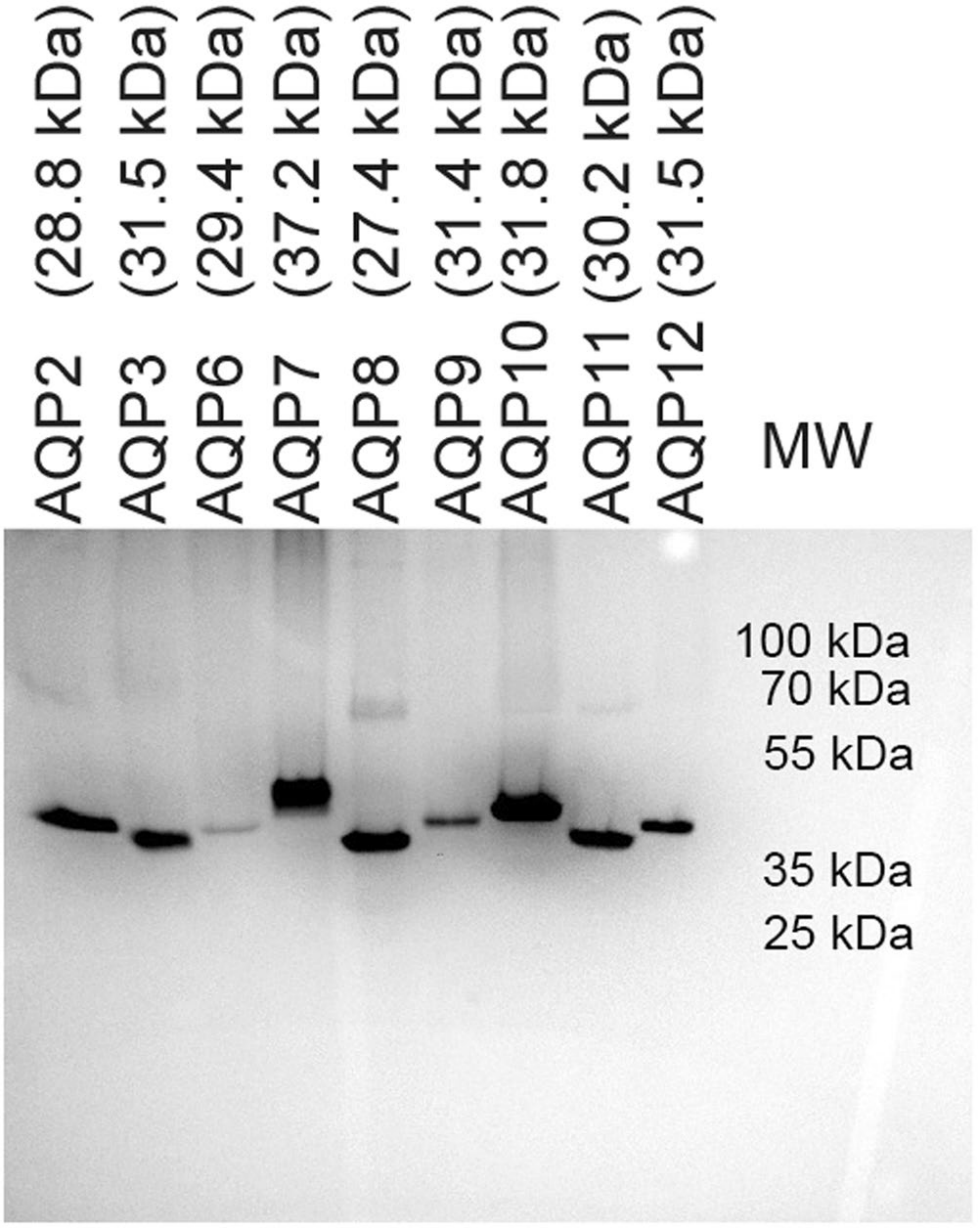
In-gel fluorescence of yeast membrane proteins. Crude membranes isolated from *S. cerevisiae* producing the indicated codon optimized human AQP-TEV-GFP-His_10_ fusion proteins at 15 °C were separated by SDS-PAGE and visualized by in-gel fluorescence. Crude membranes were isolated from yeast harvested at the time after induction that showed the highest fluorescence. 25 µg membrane protein was applied to each lane of the gel. All samples were separated in the same gel and exposed collectively.

Data in Fig. 2 showed that accumulation of fluorescence in crude yeast membranes were maximal at 15 °C. To determine if the human AQPs accumulated as full length proteins in a stable form, crude yeast membranes were separated by SDS-PAGE and analyzed by in-gel fluorescence. The results in Fig. 3 demonstrate that all AQPs accumulated in a stable form in the yeast membranes as no fluorescent degradation products were visible. It can also be seen that the electrophoretic mobility of each AQP matched their molecular weight fairly well. Only exceptions are AQP2 and AQP3 that showed a slightly higher and lower mobility, respectively, than expected. Aberrant mobility in SDS-PAGE is commonly observed for membrane proteins^37^. It is also evident from Fig. 3 that AQP7 and AQP10 accumulated in two different forms that may result from post-translational modifications.

### Production temperature dictates the degree of AQP mal-folding

**Figure 4:**
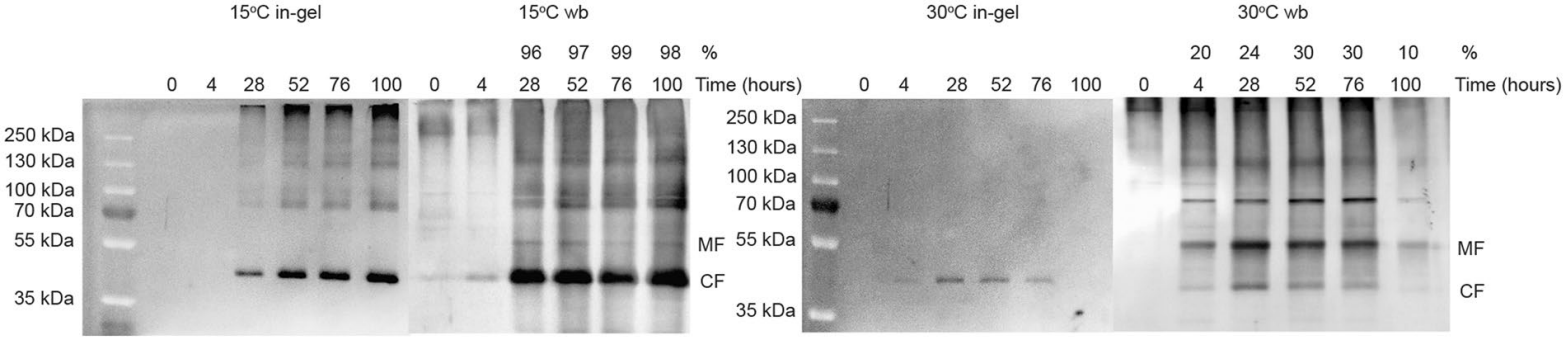
Temperature dependent folding of AQP2-TEV-GFP-His_10_. An AQP2 expressing yeast strain was grown at room temperature until OD_450_ = 1.0 and the culture was divided in two. One portion was transferred to 15 °C and the other was transferred to 30 °C. After thermo-equilibration AQP2 production was initiated by addition of 2% galactose. Crude membranes were isolated from cells induced for 0, 4, 28, 52, 76 and 100 hours. From each time point and both temperatures 25 µg crude membranes were separated by SDS-PAGE and AQP2-TEV-GFP-His_10_ was visualized by in-gel fluorescence. Samples for in-gel fluorescence were separated in two SDS-PAGE gels; one for the 15 °C samples the other for the 30 °C samples. The two gels were exposed simultaneously to visualize the GFP fluorescence. After fluorescence detection the SDS-PAGE separated membrane proteins were blotted onto a PVDF membrane and AQP2-TEV-GFP-His_10_ was visualized with an anti-GFP-antibody. Like for in-gel fluorescence the western blots were imaged simultaneously. CF, correctly folded AQP2-TEV-GFP-His_10_ monomer; MF, mal-folded AQP2-TEV-GFP-His_10_; %, percent correctly folded AQP2-TEV-GFP-His_10_.

To investigate the temperature dependent accumulation demonstrated in Fig. 2, we took advantage of the chemical properties of GFP. It was previously shown in *E. coli* that a C-terminal GFP tag only folds correctly *in vivo* if its membrane embedded fusion partner folds correctly in the plasma membrane^38^. This folding assay is based on the observations that GFP fluorescence is preserved after SDS-PAGE and correctly folded GFP only contributes with 10–15 kDa to the molecular weight of its denatured AQP fusion partner. In contrast fully denatured GFP increases the molecular weight with 28 kDa. Therefore, only correctly folded GFP can be detected by in-gel fluorescence while both correctly folded and mal-folded GFP are easily visualized by western blotting due to their rather large difference in electrophoretic mobility. As an example we analyzed the AQP2 content in crude membranes by in-gel fluorescence and subsequent western blotting with an anti-GFP-antibody. Two important issues are apparent from Fig. 4; (i) recombinant AQP2 does accumulate at both temperatures and ii) at 15 °C between 96 and 98% of AQP2-GFP fusion was fluorescent and presumably correctly folded while at 30 °C only 10 to 30% was fluorescent and therefore probably correctly folded. So folding of AQP2 in the heterologous yeast host seems to be compromised by a production temperature of 30 °C.

### Recombinant human AQPs localize differently in *S. cerevisiae*

**Figure 5:**
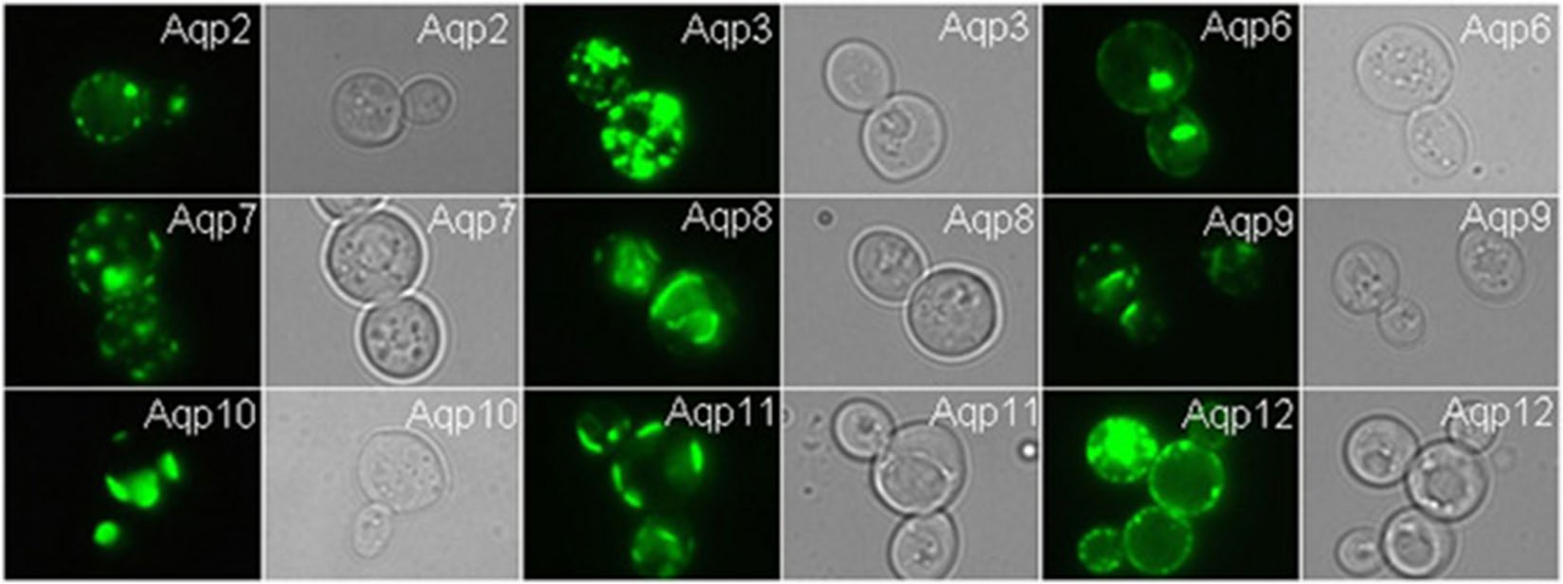
Live cell bio-imaging of yeast cells producing AQP-TEV-GFP-His_10_ fusions. Yeast cells were grown at room temperature in expression medium and induced for recombinant AQP production at 15 °C for 48 hours as described in Methods. Pairwise GFP and DIC images are shown.

In human each AQP is expressed in a tissue specific manner but also in different cellular compartments^39^. The intracellular location of some of the AQPs is furthermore regulated by post translational modifications^39^. We applied live cell bioimaging of *S. cerevisiae* producing the individual AQP-TEV-GFP-His_10_ fusions at 15 °C to discriminate between localization in the plasma membrane and in intra-cellular compartments. The data in Fig. 5 show that while AQP12 seems to accumulate primarily in the plasma membrane, AQP2, 6, 7 and 9 only partly accumulated in the plasma membrane and AQP3, 8, 10 and 11 localized entirely to internal membranes.

### Recombinant human AQP7, 10 and 12 are glycosylated

**Figure 6:**
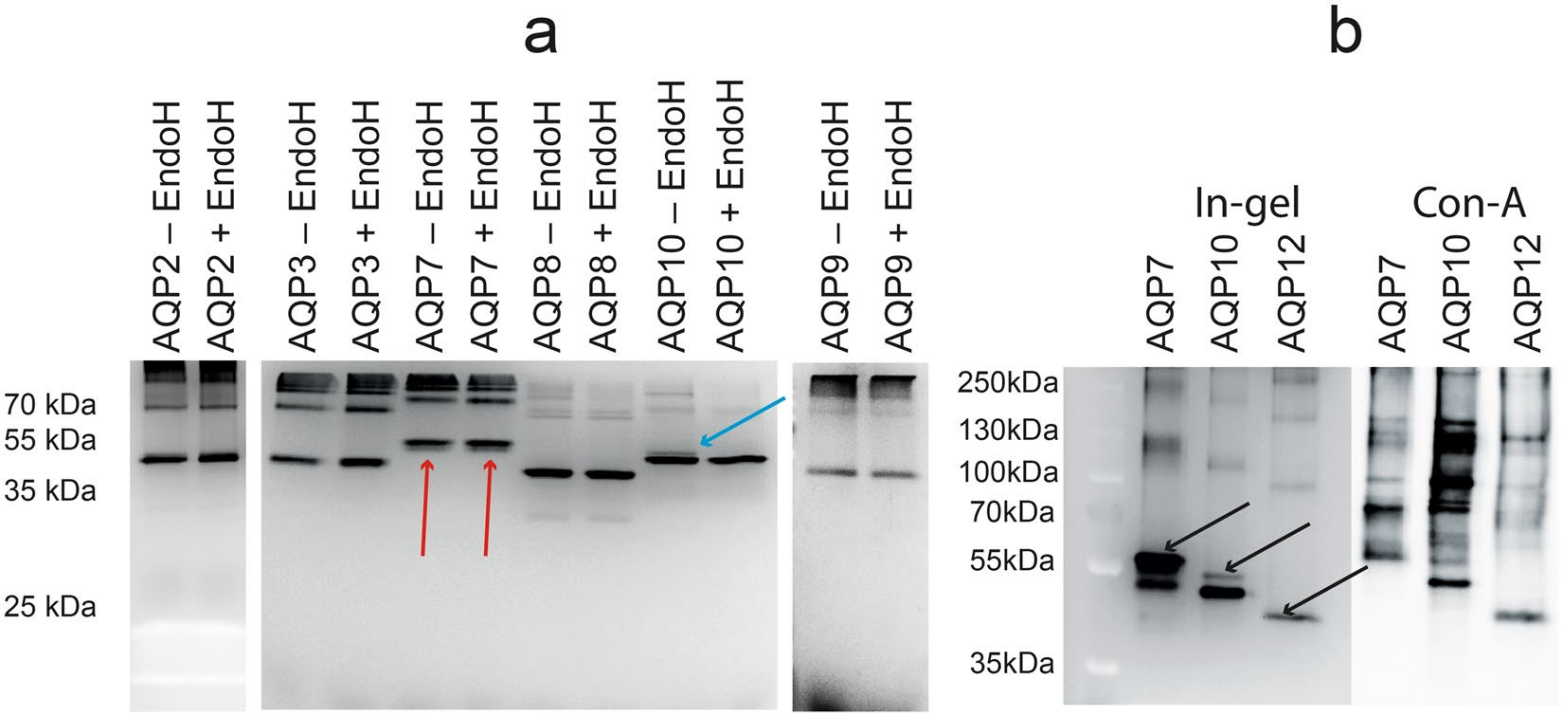
Human AQP 7, 10 and 12 are glycosylated in yeast. (**a**) crude membranes from yeast expressing the indicated human AQPs were treated with Endo-H over night as described in Methods. Endo-H (+Endo-H) and untreated (- Endo-H) samples were separated by SDS-PAGE and analyzed by in-gel fluorescence. The samples were separated in the three different gels indicated. Gels were not exposed simultaneously. A red arrow indicates a minor fraction of the AQP7 protein with a higher electrophoretic mobility. The blue arrow shows the N-glycosylated AQP10 protein band that is sensitive to Endo-H treatment. (**b**) IMAC purified AQP 7, 10 and 12 were analyzed by in-gel fluorescence and western blotting using Horse-radish Peroxidase conjugated Concanavalin A that binds mannose residues. Arrows indicate AQP proteins that contain mannose residues. The gels in “**a**” have not been cropped while the blot in “**b**” was cropped to exclude the part of gel containing the small molecular weight proteins; the removed part of the blot did not reveal any bands.

Bioinformatics predicts that AQP2, 3, 8, 9 and 10 are N-glycosylated in humans^40^ and AQP7, 10 and 12 are O-glycosylated^41^. The predicted glycosylation sites are shown in Supplementary Figure S3. To clarify if the recombinant AQPs produced in our platform are N-glycosylated we used in-gel fluorescence to analyze the electrophoretic mobility of the GFP-His_10_ tagged AQPs before and after treatment with Endo-glycosidase H. It can be seen from Fig. 6 that recombinant AQP10 is the only N-glycosylated AQP as the mobility of the slowest migrating very faint AQP10 band was the only one affected by Endo-H treatment. From the in-gel fluorescence we estimated that the N-glycosylated band represents around 1 percent of the total amount of AQP10. To determine whether AQP7, 10 and 12 are O-glycosylated we performed a western blot with Horse Radish Peroxidase conjugated Concanavalin A (ConA) that binds the mannose residues found in glycosylated yeast proteins. The data in Fig. 6 show that only the slowest migrating AQP7 band was recognized by ConA and therefore represents an O-glycosylated version of this AQP while the fastest migrating band is not glycosylated as it does not bind ConA. Comparing the in-gel fluorescence and the ConA western blot in Fig. 6 shows that ConA specifically recognizes the N-glycosylated version of AQP10. AQP12 was also recognized by ConA indicating that this protein accumulates in an O-glycosylated form in our yeast production platform.

### Recombinant human AQPs show different solubilization profiles

**Figure 7:**
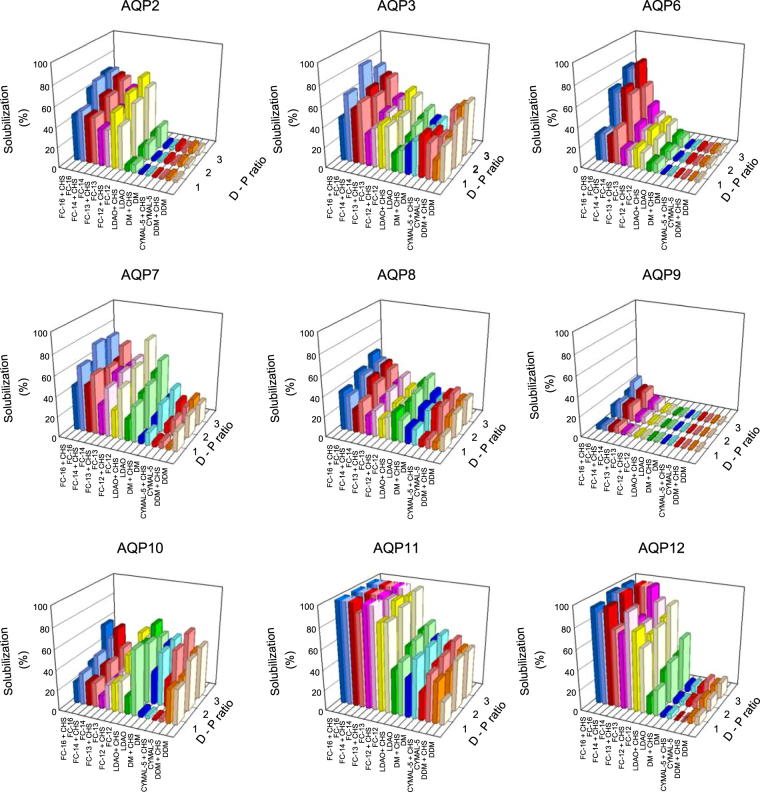
Detergent screen for recombinant human AQPs. AQPs were solubilized as described in Methods at the indicated detergent to protein ratios (w/w) (D-P ratio). Solubilization was performed in presence and absence of cholesteryl-hemi-succinate. The detergent to cholesteryl-hemi-succinate ratio was three to one (w/w). Abbreviations; FC, Fos-Choline; Cymal-5, 5-Cyclohexyl-1-pentyl-β-D-maltoside; DM, n-Decyl-β-D-maltopyranoside; DDM, n-Dodecyl-β-D-maltopyranoside; LDAO, lauryldimethylamine N-oxide; CHS, cholesteryl-hemi-succinate. Solubilization was estimated as GFP fluorescence of the solubilized protein relative to the GFP fluorescence in the non-solubilized membranes.

We set up a screen with eight different detergents to identify conditions that efficiently solubilize each of the recombinant human AQPs. Based on previous experience we performed the screen in presence and absence of cholesteryl-hemi-succinate^32,33^ at three different detergent-protein ratios. Data in Fig. 7 show that solubilization profiles and efficiencies varied significantly among the different AQPs. AQP9 stands out as it resisted solubilization in almost all tested detergents, as the maximal solubilization amounted to less than thirty percent and was obtained with the zwitter ionic detergents FC-14 and FC-16. For each of the other AQPs we identified several detergents that showed solubilization efficiencies of at least 50%. In general the super-AQPs, AQP11 and AQP12, showed the highest solubilization efficiency of all AQPs. This was achieved in the Fos-cholines and LDAO zwitter ionic detergents. The Fos-cholines solubilized almost one hundred percent of AQP11 and more that eighty percent of AQP12, while the solubilization efficiency for LDAO was around sixty percent. A significant difference between the two super-AQPs is that the non-ionic detergents solubilized around fifty percent of AQP11 but less than twenty percent of AQP12. The solubilization profiles of AQP2 and AQP6 resemble that of AQP12 as only the zwitter ionic detergents were able to solubilize more than 50% of this AQP while the non-ionic detergents solubilized less than 20%. AQPs 3, 7 and 8 showed more complex solubilization profiles. The highest solubilization efficiencies for all of these AQPs were achieved in the zwitter ionic Fos-cholines but some of the non-ionic detergents also solubilized well. DDM for example solubilized almost 50% of AQP3 while DM was the most efficient non-ionic detergent for AQP7 and AQP8. AQP10 was in a class of its own as the solubilization efficiencies for the non-ionic detergents were in the same range as for the zwitter ionic detergents. The detergent screen depicted in Fig. 7 also revealed that irrespective of the AQP and detergent presence of cholesterol did not significantly affect solubilization efficiencies.

### The homogeneity of solubilized AQP is detergent specific and cholesterol dependent

**Figure 8:**
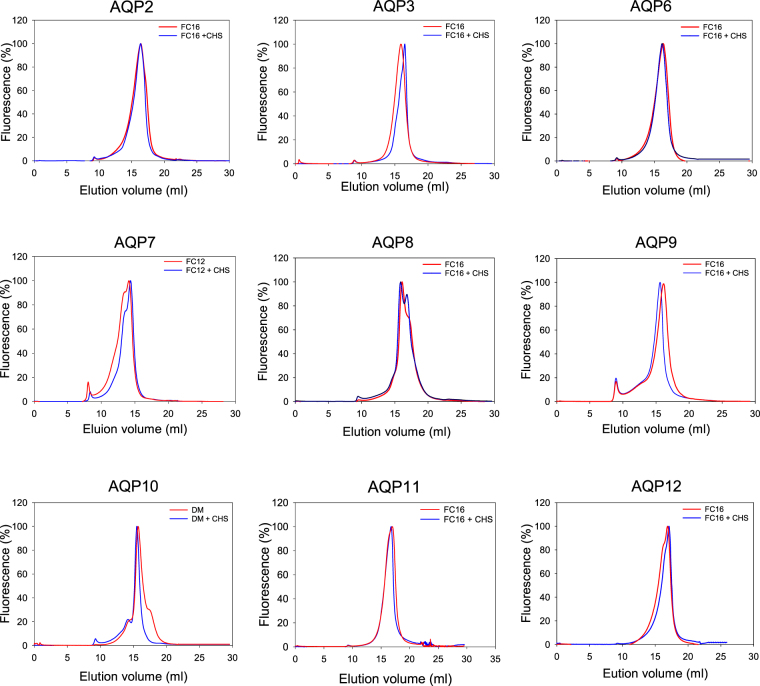
Fluorescence-detection size exclusion chromatography of solubilized AQPs. Fluorescence-detection size exclusion chromatography was used to evaluate the quality of detergent solubilized AQPs. Detergent solubilized membrane proteins were separated on a Superose 6 increase 200 10/300 GL column as described in Methods. The figure shows the FSEC profiles for the detergents used for purification and for the same detergents supplemented with Cholesteryl hemi-succinate (CHS). All FSEC profiles can be found in the Supplementary Figure S4. Fluorescence has been normalized to the peak value in each profile. The void volume is around 8 ml.

A high yield of pure, recombinant AQP requires a detergent that causes efficient solubilization and results in a stable, homogenous and active AQP preparation. We used fluorescence-detection size exclusion chromatography (FSEC) to evaluate the homogeneity of each AQP solubilized in either pure detergent or in a detergent cholesterol mixture. The FSEC analysis was performed for each detergent that solubilized more that 20 percent of the membrane embedded AQP. The FSEC profiles obtained for the orthodox AQPs, the aquaglyceroporins and the super-AQPs are shown in Supplementary Figure S4, while the FSEC profiles in detergents used for purification of each AQP are shown in Fig. 8. The orthodox AQPs (AQP2, 6 and 8) were most efficiently solubilized by the zwitterionic detergents but AQP8 was also solubilized to some extend in the non-ionic detergents (Fig. 7). It can be seen from Fig. 8 that the FSEC profile of AQP2 was unaffected by the presence of cholesterol and that the mono-dispersity was prominent in all the tested Fos-cholines but FC-14 and FC-16 seem to give the most symmetrical elution profiles. The FSEC profiles of AQP6 is seen from Fig. 8 and Supplementary Figure S4 to rely more on the detergent used for solubilization, as only FC-16 resulted in a mono-disperse elution profile. The elution profile was the same in detergent alone as in detergent with cholesterol. In contrast to the situation with AQP2 and AQP6 the mono-dispersity of AQP8 was not very good in the zwitter ionic detergent FC-14 and FC-16. In fact these detergents seem to interfere strongly with protein-protein interactions as the elution profiles showed two peaks indicating partly disassembly of the AQP tetramer. This effect seems to be enhanced by the presence of cholesterol. However, the quality of AQP6 solubilized in non-ionic detergents DM, DDM and CY-5 was positively affected by the presence of cholesterol during solubilization as presence of CHS gave rise to excellent symmetrical elution profiles.

### Ni-affinity chromatography results in almost pure AQP preparations

**Figure 9:**
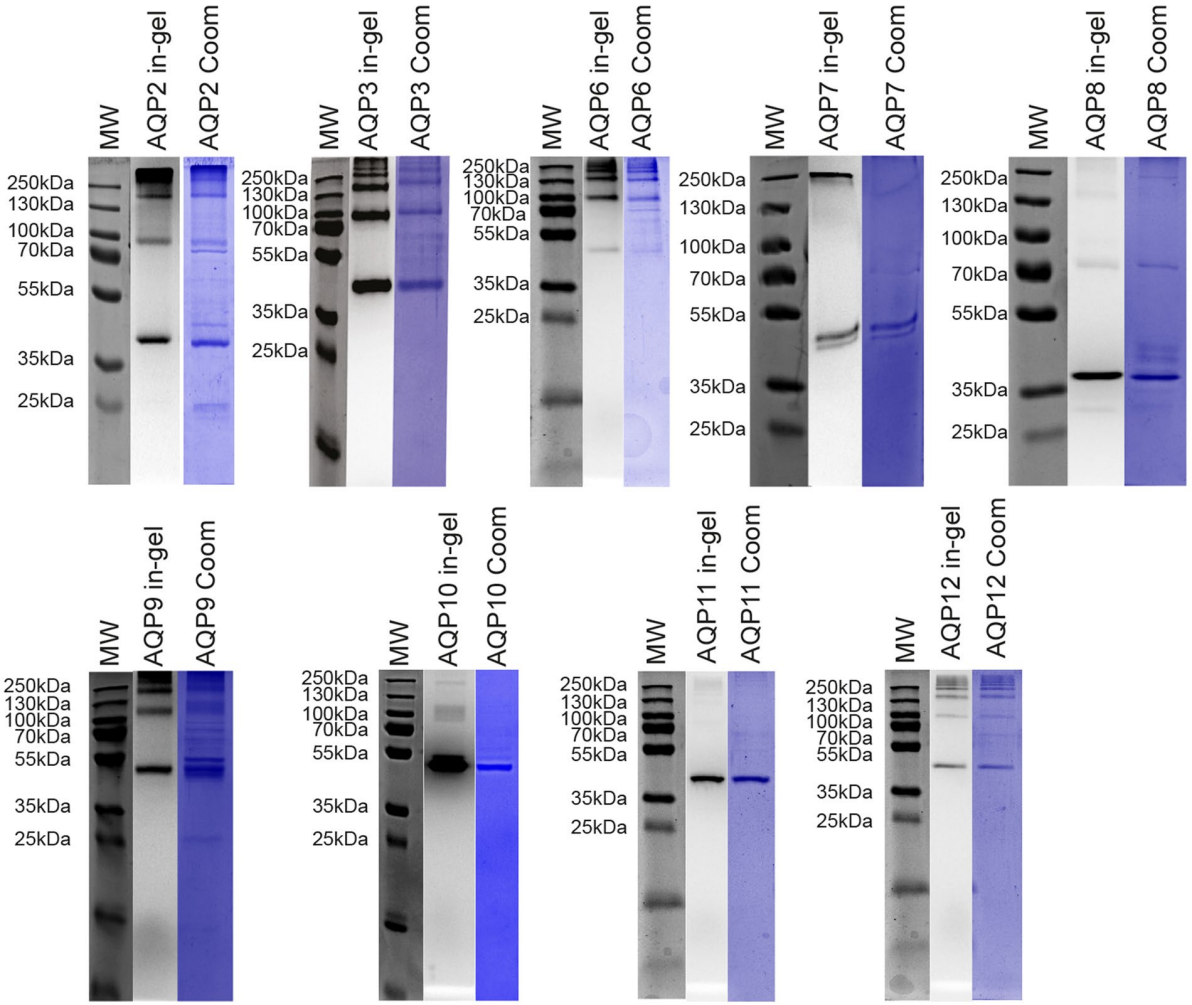
SDS-PAGE of purified AQPs. Based on the elution profile from Supplementary Figure S5, fractions containing the highest amount of fluorescence were analyzed by SDS-PAGE. AQP-TEV-GFP-His_10_ fusions were visualized by in-gel fluorescence and the gel was subsequently stained with Coomassie to visualize all proteins present in the purified sample. Abbreviations; MW, a molecular weight marker; in-gel, in-gel fluorescence; Coom, Coomassie stained gel.

For purification of each AQP we selected a detergent that solubilized well and showed a monodisperse FSEC elution profile (Fig. 8). The elution profile observed for each AQP during Ni-affinity chromatography is shown in Supplementary Figure S5. It is evident from these data that the AQPs show different elution profiles although they all carry the same TEV-GFP-His_10_ tag. To determine the purity of each AQP we separated the peak fractions by SDS-PAGE and visualized the AQP content by in-gel fluorescence and subsequently Coomassie staining. The data in Fig. 9 show that the protein purity after Ni-affinity chromatography was very high as no or only few non-fluorescent bands with minor intensities were visible after Coomassie staining. It is also evident from Fig. 9 that the resistance of the tetrameric structure to SDS varies among the nine AQP; for AQPs 7, 8, 10 and 11 the monomer is the only visible band while for AQP2, 3, 6, 9 and 12 the dimer, trimer and tetramer are visible, too. AQP6 stands out as the only one where the monomer is not the most prominent band after SDS-PAGE.

### TEV cleavage releases the AQP to various degrees

**Figure 10:**
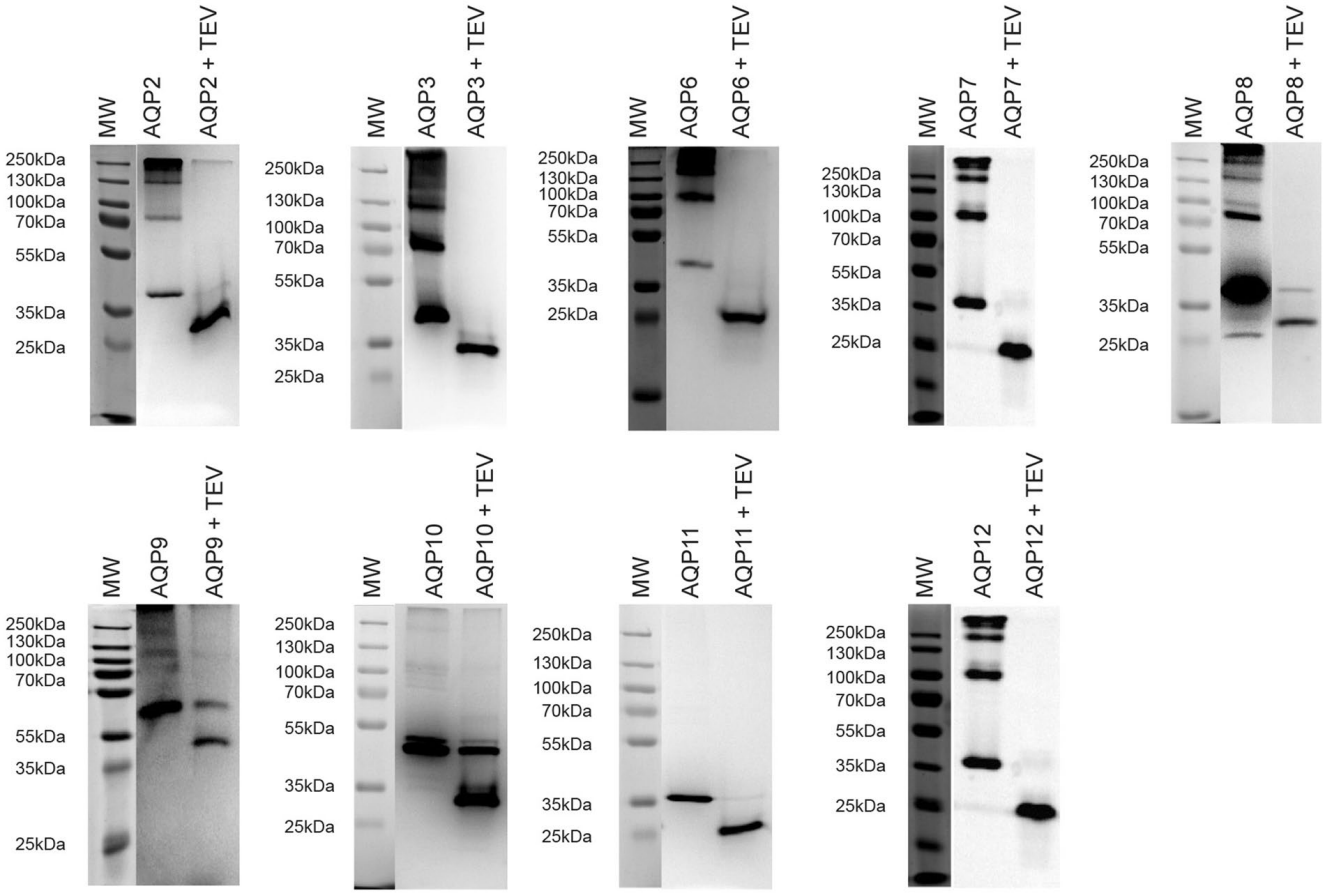
TEV digestion of purified AQP-TEV-GFP-His_10_ fusions. The purified AQP-TEV-GFP -His_10_ fusions from Fig. 9 were incubated with TEV protease over night at 4 °C as described in Methods. The outcome of each TEV digestion and a non-digested sample were separated by SDS-PAGE and visualized by in-gel fluorescence. Abbreviations; MW, molecular weight marker.

Peak fractions from the Ni-affinity purification shown in Fig. 9 were treated with TEV protease and subsequently separated by SDS-PAGE along with an undigested sample. The data in Fig. 10 show that except for AQP9 and AQP10 TEV protease digestion was almost complete.

### Purified AQPs are active after reconstitution into polymersomes

Comparison of AQP permeabilities has mostly been based on results obtained in intact cells like *Xenopus* oocytes^42^ and yeast cells^43^. Although much valuable information has been gathered the *in vivo* analysis is complicated by the fact that the recombinant AQPs are assumed to be located to the same extent in the plasma membrane. As an approach to circumvent this we investigated water and glycerol permeability after reconstitution of purified AQPs into proteopolymersomes using stopped flow kinetics. Each AQP was reconstituted into separate proteopolymersomes using the diblock copolymer PDMS-PMOXA as previously described^44^. In short, PDMS-PMOXA self-assembles into polymersomes in the absence of protein and into proteopolymersomes in the presence of detergent solubilized membrane protein. We used this method to create a library of separately reconstituted human AQPs, which were then extruded through a 200 nm filter. Dynamic light scattering (DLS) was used to measure the hydrodynamic diameter of the proteopolymersomes and the blank polymersomes. The proteopolymersomes with human AQPs were all measured to be in the range of 110 ± 8 nm to 162 ± 14 nm (Table 1). This is comparable to blank polymersomes which yielded polymersomes with a hydrodynamic diameter of 121 ± 5 nm. This indicates a comparable self-assembly process and suggests that the presence of membrane protein did not influence the formation of polymersomes and hence that they reconstituted successfully. Since each AQP is reconstituted under the same conditions as well as with the same detergent and polymer matrix, we can compare water as well as glycerol transport, and to the best of our knowledge no such comparative study has been described before.

**Table 1:** Activity of purified and reconstituted human AQPs.

AQP	Hydrodynamic diameter (nm)	k_i_ (s^−1^)	R (%)	*P*_*f*_ (10^–6^ m/s)	Δdia (nm)	Δk_i_ (s^−1^)
Empty	121 ± 5	200	99.6	624 ± 26	—	—
APQ2-GFP	135 ± 8	1784	99.6	6212 ± 83	−17	1
AQP3-GFP	162 ± 14	2111	99.5	8820 ± 773	119	1122
AQP6-GFP	114 ± 4	769	99.7	2261 ± 82	−4	12
AQP7-GFP	110 ± 8	715	99.7	2029 ± 151	2	171
AQP8-GFP	123 ± 13	2065	99.5	6551 ± 701	−8	3
AQP9-GFP	156 ± 14	2052	99.5	8256 ± 751	14	782
AQP10-GFP	115 ± 4	1899	99.7	5624 ± 195	1	121
AQP11-GFP	120 ± 7	844	99.7	2612 ± 156	4	498
AQP12-GFP	135 ± 11	2066	99.5	7194 ± 595	5	1086
AQP10His_8_	102 ± 15	2056	99.7	5453 ± 742	32	892

The second column shows the hydrodynamic diameter measured by DLS. Column three shows k_i_-values as determined by fitting the light scattering signal to a second order exponential function. Column four shows the R-value for the fit to the experimental data. Column five shows the calculated water permeability (*P*_*f*_) of each human AQP. Column six shows the change in diameter (Δdia) measured by DLS after incubation with glycerol. Column seven shows the change in k_i_-values (Δk_i_) due to incubation with glycerol.

### All recombinant human AQPs mediate a water flux

After reconstitution into proteopolymersomes we used stopped-flow to measure the separating properties of human AQPs both for water and glycerol. Representative data for the normalized light scattering are shown in Supplementary Figure S6 where NaCl was used as a draw solute. The light scattering intensity of blank control polymersomes indicates a very slow shrinkage and hence very slow water permeability under the applied osmotic pressure. The reconstituted human AQPs show a higher light scattering intensity under identical applied osmotic pressure. This indicates a much faster shrinkage of the proteopolymersomes and hence a higher water permeability. By fitting the data to a double exponential function the faster kinetic rate constant k_i_ (s^−1^) of the water flux can be determined as the rate constant of AQP mediated water transport. This rate constant is directly proportional to the water flux out of the proteopolymersomes. The k_i_-values listed in Table 1 suggest that all the reconstituted AQPs are active and permeable to water. To further compare water permeation through the recombinant AQPs we calculated the osmotic water permeability of the proteopolymermembrane, *P*_*f*_, by the following equation^45^:

1 $${P}_{f}=\,\frac{{k}_{i}}{\frac{S}{{V}_{0}}\,\ast \,{V}_{w}\,\ast \,{\rm{\Delta }}Osm}$$

where k_i_ is the experimentally determined kinetic rate constant, S/V_0_ is the surface to volume ratio, V_w_ is the partial molar volume of water (0.018 L/mol) and $\triangle$ Osm is the difference in osmolarity between the intra- and extracellular aqueous solutions calculated with the theoretical values for NaCl and PBS buffer. The stopped-flow was performed by mixing equal amounts of NaCl with proteopolymersome-solution giving an osmolarity difference of 359.5 mOsm//L across the proteopolymersomes. Since each AQP is stabilized by the same detergent (LDAO) and reconstituted by an identical polymer matrix under identical conditions we assume identical polymer-to-protein ratios after reconstitution. Hence we can compare the water permeabilities to compare the water transport abilities between the AQPs. The calculated *P*_*f*_ values are listed in Table 1 and show a remarkable increase in *P*_*f*_ compared to the blank control polymersomes.

### AQP3, 7, 9, 10, 11, 12 mediate a glycerol flux

To demonstrate glycerol transport, the proteopolymersomes and blank polymersomes were incubated with glycerol to mediate glycerol transport into the proteopolymersomes. This strategy has previously been described^45^. The hydrodynamic diameter of the polymersomes was measured with DLS before and after incubation with glycerol. The difference in diameter (Δdia) before and after glycerol incubation is shown in Table 1. Negative values indicate reduction of diameter, while positive values indicate increase in diameter. As seen from the data, proteopolymersomes with reconstituted AQP3, 7, 9, 10, 11 and 12 were swelling. We assign the swelling to permeability of glycerol into the proteoploymersomes. For proteopolymersomes with reconstituted AQP2, 6 and 8 we observe small shrinkage which we assign to permeability of water out of the proteopolymersome, due to the difference in osmotic pressure across the polymersome membrane.

To further verify activity of the AQPs, we compared stopped-flow measurements of the glycerol incubated proteopolymersomes to identical proteopolymersomes without incubation. After overnight incubation with glycerol we assumed establishment of an osmolaric equilibrium across the polymer membrane. Exposing the proteopolymersomes to a NaCl solution of identical osmolality we could measure water transport across the membrane if no glycerol had been transported into the proteopolymersome. The water transport properties were evaluated by stopped-flow measurements. Typical measurements of polymersomes and proteopolymersomes are shown in Supplementary Figure S7 using NaCl as a draw solute. For reconstituted AQP2, 6, and 8 the k_i_ (s^−1^) of water transport was comparable to that measured before glycerol incubation indicating that there was no uptake of glycerol during the incubation time. For reconstituted AQP3, 7, 9, 10, 11 and 12 however the measured k_i_ (s^−1^) was remarkably reduced after incubation with glycerol, indicating that the AQPs were transporting glycerol into the proteopolymersomes. The numerical value of the change in k_i_ before and after incubation with glycerol is shown in Table 1 as Δk_i_.

In conclusion, we have shown that all studied AQPs mediate water permeation while all except AQP2, 6 and 8 also allow a glycerol flux. Our experiments suggest that under identical conditions the water permeation of AQP3 > AQP9 > AQP12 > AQP8 > AQP2 > AQP10 > AQP11 > AQP6 > AQP7 (Table 1). To our knowledge this is the first time that such a large number of AQPs have been analyzed in the same experimental setup.

### Large scale production and purification of AQP10-His_8_

**Figure 11:**
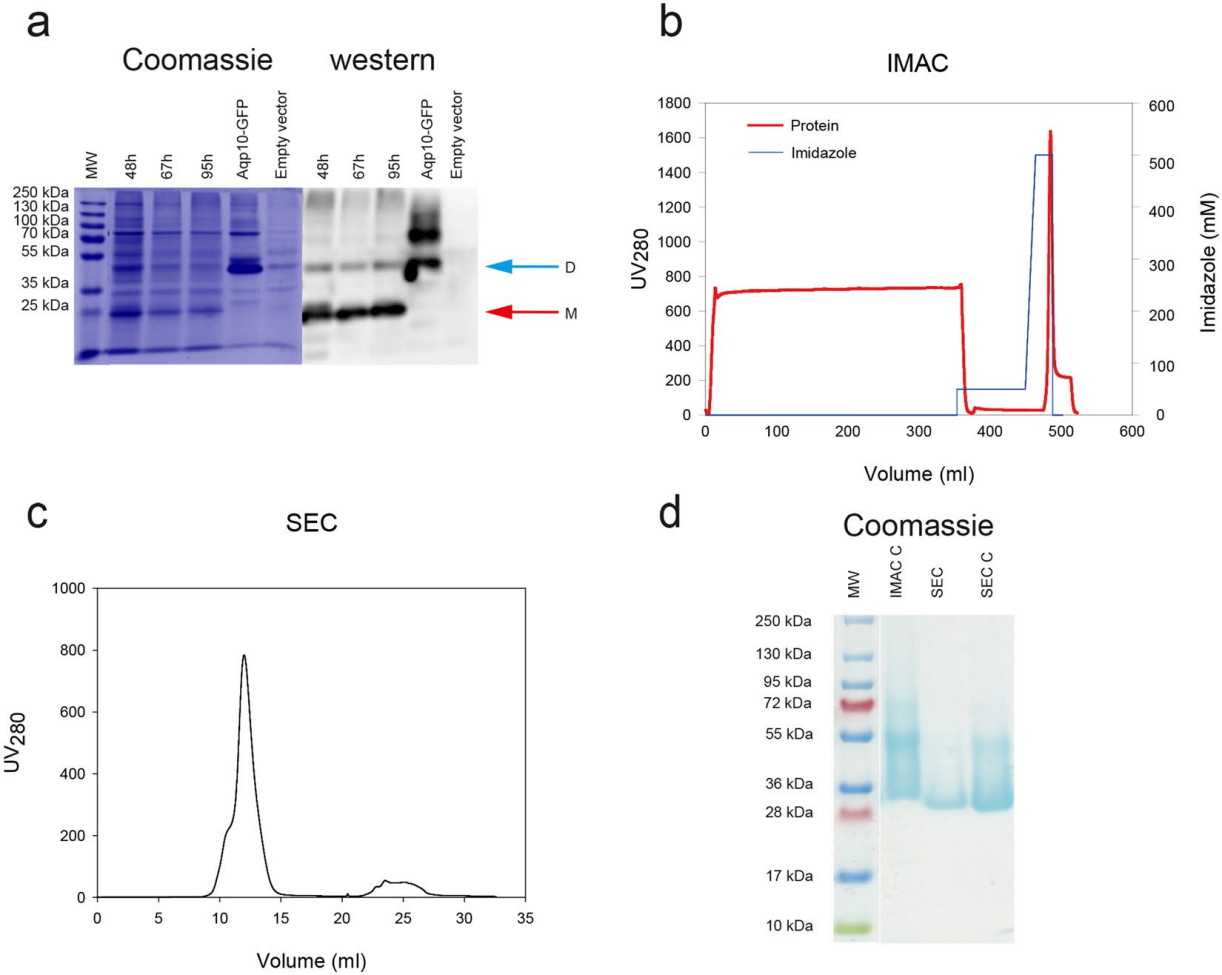
Large scale expression and purification of AQP10-His_8_. (**a**) Accumulation profile for AQP10-His_8_ in membranes from *S. cerevisiae* grown at 15 °C in a computer controlled 15-L bioreactor and induced for expression for the indicated periods of time. AQP10-His_8_ was visualized by Coomassie staining or western blotting after SDS-PAGE separation of crude membranes. A membrane sample from AQP10-TEV-GFP-His_10_ expressing cells was included as positive control and a membrane sample from empty vector-transformed cells was used as a negative control. Arrows indicate monomeric (M, red) and dimeric (D, blue) form of AQP10-His_8_, respectively. (**b**) Immobilized metal affinity chromatography (IMAC) profile of large-scale AQP10-His_8_ purification as described in Methods. Briefly, membrane proteins were solubilized in DM and bound protein (red line) was competitively eluted by a linear imidazole gradient (50-500 mM; blue line) in 20 mM Tris pH 7.5, 200 mM NaCl, 20% glycerol, 5 mM β-ME and 1 (w/v)% bOG. (**c**) Size exclusion chromatography (SEC) elution profile of concentrated IMAC-purified sample. SEC was performed using a Superdex increase 200 10/300 GL column in 20 mM Tris pH 7.5, 100 mM NaCl, 10% glycerol, 2 mM β-ME and 1% bOG. (**d**) SDS–PAGE gel (Coomassie staining) following large-scale AQP10-His_8_ purification. IMAC C: concentrated IMAC-purified sample, SEC: SEC peak fraction, SEC C: concentrated SEC-purified sample.

Based on the successful production and purification of all human AQPs with preservation of activity, we decided to determine if our platform is useful for large-scale production of AQPs. As an example we analyzed expression and purification of non-GFP tagged AQP10-His_8_, in a 15-L bioreactor setup. To identify the optimal time point for cell harvest we analyzed the kinetics of AQP10-His_8_ accumulation. The data in Fig. 11 reveal that already 48 h post induction, AQP10-His_8_ was visible in crude membranes mainly as intense bands representing monomeric (M) and dimeric (D) forms. As evident from the immunoblot, accumulated peaked around 48 hours and then leveled off slowly, indicating the existence of a rather large time window for harvesting the cells without compromising the yield. It also appears from Fig. 11a that the expression level of AQP10-His_8_ was approximately the same as for the GFP fusion. For large-scale protein purification we used 50 g of cells harvested 95 hours post-induction (Fig. 11b). Based on the detergent screen in Fig. 7 crude yeast membranes were solubilized in DM, subjected to IMAC and AQP10-His_8_ was eluted in buffer containing bOG to exchange the detergent. Immobilized metal affinity purification (Fig. 11b) yielded approximately 26.4 mg of higly pure AQP10-His_8_ protein (Fig. 11d; lane IMAC C). Subsequent SEC analysis of the IMAC-purified sample revealed that the purified protein was homogenous in bOG and devoid of aggregation (Fig. 11c), as reflected by the monodisperse shape of the main peak and lack of signal in the void volume, respectively. Furthermore, the final purity of the SEC-grade sample was high (Fig. 11d; lanes SEC and SEC c). In addition we showed that the purified AQP10-His_8_ protein was able to mediate a water flux and a glycerol flux similar to that of the GFP tagged version after reconstitution into polymersomes (Table 1). It can therefore be concluded that our production platform successfully overproduced a high-quality protein sample suitable for biophysical studies.

## Discussion

The present paper addresses one of the issues that seriously restricts membrane protein characterization; lack of access to large amounts of prime quality human membrane proteins. Establishment of a standardized, cheap and easily handled platform for high yield recombinant production and purification of human membrane proteins would significantly improve our ability to study membrane proteins by means of biochemical and biophysical techniques and increase the rate by which membrane protein structures are determined.

The focus of the present paper therefore was to explore how well our *Saccharomyces cerevisiae* platform^30^ matches these requirements. As model proteins we investigated production and purification of nine out of the thirteen AQPs found in humans. AQP3, 6, 7, 8, 9, 10, 11 and 12 were selected as no high-resolution structure is available for these AQPs. AQP2 was included for reasons of comparison as it has previously been categorized as a difficult-to-express protein^34,35^.

The methylotrophic yeast *Picha pastoris* has previously been exploited for its capacity to produce all human AQPs^34,35^. A major conclusion from these studies was that although the human AQPs are very homologous, large variations were observed in the expression levels. We used the results obtained in *P. pastoris* to benchmark the capacity of our *S. cerevisiae* platform for AQP production. Out of the nine AQPs analyzed in the present study, only AQP3, AQP7, AQP10 and AQP12 were expressed in *P. pastoris* at a level compatible with purification, while AQP2 and AQP8 expression was near the threshold for useful overproduction. AQP6, AQP9 and AQP11 resulted in levels below the detection level in *P. pastoris*. The membrane densities obtained for the various human AQPs produced in *Pichia* amounted to between 0 and 0.7% of the total membrane protein content^35^. This is in contrast to the results presented in the present paper as the nine AQPs accumulated at membrane densities between 0.9% and 8% of total membrane protein content (Fig. 2). The major advantage of using *P. pastoris* as an expression host is its ability to grow to an exceptional high cell density resulting in a very large amount of recombinant AQP per liter. However, for non-secreted proteins like AQPs the amount that can be produced per liter is not as relevant as the amount that accumulates per cell. The high density of membrane embedded AQP per cell is more important, as this simplifies purification and decreases the associated costs.

Construction of expression plasmids can be one of the most tedious steps in optimizing recombinant protein production. To ease the construction of AQP expression plasmids we exploited *Saccharomyces cerevisiae’s* unique ability to perform homologous recombination^46^. As indicated in Fig. 1 this makes construction of recombinant expression plasmids fast, precise and economically feasible as it does not require expensive enzymes but only transformation with a linearized expression plasmid and PCR products. Furthermore, this approach generates the expression plasmids directly in the yeast host used for expression and does not add any irrelevant nucleotide sequences as for instance restriction sites to the final constructs. Presence of such “contaminating” sequences has been shown to reduce recombinant protein production^47^.

As we have seen before for other recombinant membrane proteins^31–33^ expression temperature was very critical for obtaining a high yield of AQP. As evident from Fig. 2 all fluorescent AQPs accumulated to a substantially higher membrane density at 15 °C compared to 30 °C. Depending on the AQP accumulation at 15 °C was between 2.4 to 44 times higher than that obtained at 30 °C. So at least in the present platform reduction of the expression temperature to 15 °C makes the difference between success and failure in producing active membrane proteins in the amounts required for purification.

A potential drawback of using GFP fluorescence to measure recombinant protein accumulation is that fluorescence does not necessarily reflect the accumulated amount of full length AQP-TEV-GFP-His_10_ fusions in the cell but may also include fluorescent degradation products. The observation that all AQPs produced at 15 °C accumulated with the expected molecular weight and were stable *in vivo* and during membrane preparation and purification demonstrates that degradation was not a problem in the present study (Figs 3 and 9). The measured GFP fluorescence in crude membranes therefore originates from accumulation of full length proteins.

To exploit the temperature dependent accumulation we used the C-terminal GFP tag as a folding reporter^38^. The folding assay is based on the observation that correctly folded and fluorescent GFP does not denature during SDS-PAGE due to its compact β-barrel structure and therefore only contributes 10–15 kDa to the apparent molecular weight of the AQP^38^. Non-folded and therefore non-fluorescent GFP, however, contributes around 28 kDa to the AQP molecular weight^38^. The percentage of folded AQP can thus be estimated by western blotting and the correctly folded GFP can be identified by in-gel fluorescence and amounted to ≈ 97% at 15 °C and ≈ 20% at 30 °C (Fig. 4). As apparent from Fig. 4 reduced accumulation of fluorescent AQP-TEV-GFP-His_10_ at 30 °C is therefore not due to lack of protein production at this temperature but due to inefficient protein folding. Such insufficient folding can only be visualized for a C-terminal GFP tagged protein as correctly folded AQP10-His_10_ cannot be distinguished from mal-folded AQP10-His_10_ by SDS-PAGE. The discrepancy between the optimal growth temperature of yeast which is 30 °C and the optimal temperature for AQP accumulation may be explained by the data in Fig. 4 showing that folding of the heterologous AQP2 is strongly favored by the low temperature.

The intracellular localization of AQPs in a number of human tissues is well characterized^10^ and some AQPs including AQP2, AQP7, AQP8, AQP9 and AQP10 shuttle in a regulated way between intracellular compartments and the plasma membrane^48^. In contrast AQP3 always localizes to the plasma membrane while AQP6 and the super-AQPs, AQP11 and 12 always accumulate in intracellular compartments^48^. From Fig. 5 we can see that in *S. cerevisiae* AQP2, 7 and 9 localized partly to the plasma membrane while AQP8 and 10 seem to accumulate exclusively in intracellular compartments. It can also be seen from Fig. 5 that AQP3 seems to localize entirely in intracellular membranes. The intracellular AQPs also show a non-native localization in yeast as substantial amounts of AQP6 and AQP12 localized to the plasma membrane while AQP11 does not seem to accumulate in the plasma membrane. The observed deviations between AQP localization in yeast and native cells may reflect that the protein sorting mechanisms in yeast are different from those found in mammalian cells. However, as all nine AQPs produced in yeast were functional with respect to transport (Tabel 1 and Supplementary Figure S6), the present study shows that activity of a recombinant membrane protein cannot be predicted from its compartmentalization in the host cell and localization does not reflect the amount of accumulated AQP, at least not in *S. cerevisiae*.

Hyper glycosylation is sometimes reported for recombinant proteins produced in *S. cerevisiae* ^49^. For many purposes, this poses a problem as post-translational modifications introduce heterogeneity to purified recombinant proteins and is therefore an important issue for biophysical techniques such as crystallography. Bioinformatics predicts that AQP2, 3, 7, 9 and 10 are N-glycosylated^40^ and AQP7, 10 and 12 are O-glycosylated^41^ in humans, Supplementary Figure S3. In partly agreement with these predictions, SDS-PAGE of crude membranes or purified AQP7 and AQP10 revealed two bands (Fig. 3). For AQP7 the highest molecular weight was the most intense band indicating that the great majority of this protein was post-translationally modified, while for AQP10 the most intense band was the fastest migrating band suggesting that only a very minor part of AQP10 was modified. The heaviest AQP10 band represented an N-glycosylated version as the mobility of this band was sensitive to Endo-H treatment (Fig. 6). 35% of recombinant AQP10 produced in *P. pastoris* was previously shown to be N-glycosylated^50^ so for this protein there seems to be a difference between *S. cerevisiae* and *P. pastoris*. In contrast, none of the AQP7 bands were affected by Endo-H treatment indicating that this AQP was not N-glycosylated in *S. cerevisiae*. AQP2, AQP3 and AQP9 were also not N-glycosylated as SDS-PAGE separation of crude membranes containing these AQPs only showed a single band that was not affected by Endo-H treatment (Fig. 6a). Removal of O-glycosylation is more complicated than removal of N-glycosylation^51,52^. We therefore identified purified AQP7, 10 and 12 GFP fusions by in-gel fluorescence and subsequently conducted western blotting using HRP conjugated Concanavalin-A. It is apparent from Fig. 6b that the slowest migrating band of AQP7 represents an O-glycosylated version and AQP12 was completely O-glycosylated as the single fluorescent AQP12 band was also visualized by the Con-A protein. AQP10 did not seem to be O-glycosylated as only the Endo-H sensitive band was recognized by Con-A.

The extent of AQP glycome is far from fully characterized as only AQP1, AQP3 and AQP8 have been shown to be N-glycosylated^53,54^ and O-glycosylation has to our knowledge not been investigated. As the initial step in N-and O-glycosylation is preserved among eukaryotes^55^ we expect that the AQPs glycosylated in our expression system will also be glycosylated in native tissue.

Identification of a suitable detergent for purification of a membrane protein in its active form involves screening as a single detergent that fits all membrane proteins does not exist. This is also emphasized from the detergent screen in Fig. 7. Even though the AQPs show a high degree of sequence identity and very likely overall fold they respond differently to various detergents. Even among AQPs belonging to the same sub-family we see distinct differences. For the orthodox AQP2, 6 and 8, it is interesting that the two former AQPs are only solubilized in the zwitter-ionic detergents, while AQP8 is more promiscuous with respect to the detergent and solubilized quite well in the milder non-ionic detergents, too. The solubilization profiles among the four aquaglyceroporins also turned out to be very different. While AQP9 was almost resistant to solubilization in any of the investigated detergents AQP3 and AQP10 solubilized well in zwitter-ionic as well as non-ionic detergents and AQP7 showed a strong preference for the zwitter-ionic detergents. There were also significant differences between the two super-AQPs, AQP11 and 12. Both AQPs were most efficiently solubilized in the zwitter-ionic detergents but only AQP11 solubilized quite well in the milder non-ionic detergents, too. It is interesting that supplementation of the detergent with cholesterol did not increase the solubilization efficiency in contrast to what we have seen before with other membrane proteins produced in the same platform^31–33^.

The lipid composition differs considerably among different cellular membrane compartments^56^. It is therefore tempting to relate the detergent specific solubilization efficiency to the site of AQP accumulation. AQP2, 6, 7, 9 and 12 that accumulated partly or exclusively in the plasma membrane (Fig. 5) could only be solubilized in the zwitter-ionic detergents while AQP3, 8, 10 and 11 that accumulated exclusively in internal membranes (Fig. 5) could also be solubilized in the milder non-ionic detergents. So based upon the nine tested human AQPs it appears that intracellular accumulation may be beneficial for solubilization efficiencies in the milder non-ionic detergents. Even though we do not know the exact intracellular location of recombinant AQP accumulation, we know that the lipid composition of the membranes that define the Endoplasmic Reticulum and the Golgi compartment differs considerable from that of the plasma membrane. The ER membrane displays a very low concentration of sterols and complex sphingolipids meaning that this membrane has a more loose packing of lipids. The plasma membrane on the other hand is enriched in sphingolipids and sterols that are packed at a higher density than in the internal membranes. Consequently, it is tempting to propose that the dense lipid packing in the plasma membrane may require more harsh detergents for solubilization of the recombinant AQPs.

Even though high solubilization efficiency is very desirable the most important issue is that solubilization results in a homogeneous protein preparation. This can be assayed fast and easy by Fluorescence-Detection Size Exclusion Chromatography^57^. To analyze this thoroughly we performed FSEC analysis for each AQP in all detergents giving a solubilization efficiency larger than 20%. Solubilization prior to FSEC analysis was performed both in presence and absence of Cholesteryl Hemi-Succinate (Fig. 8 and Supplementary Figure S4). Overall, for each AQP we found several detergents that resulted in monodisperse elution profiles. For purification we selected the detergent that solubilized with the highest efficiency and showed a monodisperse elution profile.

AQP2 and AQP6 could only be solubilized above 20% in the Fos-choline detergents. All FSEC profiles for AQP2 were monodisperse irrespective of the presence or absence of CHS. For AQP6 the profiles were better in presence of CHS except for Fos-choline 16 that yielded the same symmetrical monodisperse elution profile in detergent and detergent plus CHS. In particular for Fos-choline 12 and 13 monodispersity was greatly improved by the presence of CHS. AQP8 differed from the two other orthodox AQP2 and 6 by being soluble in all tested detergents, although better in the zwitter-ionic ones. The FSEC profiles in the non-ionic detergents DM, DDM and Cy-5 showed very symmetrical FSEC profiles in contrast to FC-14 and FC-16 that resolved as two peaks in the chromatogram. This may result from disassembly of the tetrameric structure. Among the aquaglyceroporins AQP3 and 10 resembled one another as they solubilized well in zwitter-ionic and non-ionic detergents, while AQP7 preferred the zwitter-ionic detergents and AQP9 was almost resistant to solubilization. AQP3 yielded monodisperse elution profiles in every tested detergent and cholesterol had a positive effect on the elution profile for all detergents. AQP10 generally gave good elution profiles in all detergents except LDAO, FC-13, FC-14 and FC-16. DM was the detergent giving the most monodisperse profile, while the others and in particular the zwitter-ionic detergents resulted in an elution profile with two peaks irrespective of the presence or absence of CHS, the only exception being FC-12 alone that also resulted in a monodisperse elution profile. The zwitter-ionic detergents were most efficient for solubilization of AQP7. All except FC-12 and FC-16 in absence of CHS gave a double peak in the elution profile indicating that the tetrameric AQP structure had disassembled. The non-ionic detergents resulted in a more mono-disperse elution profile but with a tendency to yield a shoulder in the profile. The AQP9 could only be solubilized in FC-14 and FC-16 and both detergents gave nice elution profiles in the FSEC analysis. Presence of CHS improved monodispersity. AQP11 showed a monodisperse elution profile in every detergent tested except LDAO and for most detergents CHS improved the elution profile. Only DM seemed to have a negative effect on homogeneity as two peaks were observed, and this was prevented by adding CHS to the detergent. Decent solubilization of AQP12 was only achieved when using the zwitter-ionic detergents. Fortunately, the FSEC profiles in fos-cholines were monodisperse but also DDM gave a monodisperse profile that was affected by the presence of CHS.

All nine AQPs were isolated by Ni-affinity chromatography to high purity in the detergent showing the highest solubilization efficiency and most monodisperse elution profile. It is evident from Fig. 9 that all proteins were reasonably pure after affinity chromatography. The only exception was AQP9 that showed a larger amount of contaminating bands; a fact that may reflect the resistance of this AQP to solubilization. It is also evident from Fig. 9 that the stability of the detergent solubilized AQP tetramer towards SDS differed among the nine AQP. For AQP2, 3, 8, 9 and 12 the monomer was the most dominant protein but dimers, trimers and tetramers were visible, too, while for AQP7, 10 and 11 the monomer was almost exclusively the only visible band. AQP6 was different in the sense that the monomeric band was hardly visible while the dimer, trimer and tetramer dominated. So the stability of detergent solubilized AQPs as judged by SDS-PAGE appear to vary substantially.

Presence of the GFP tag is a considerable advantage for optimization of expressing and purification. Nevertheless, fusion forms may however be disadvantageous for various biophysical characterization techniques including crystallography. The TEV site separating each AQP and the GFP-His_10_ tag was cleavable for most of the proteins (Fig. 10). However, the in-gel fluorescence assay revealed that the AQP9 and 10 fusions were not digested to completion and also the AQP8 fusion showed a substantial amount of non-digested protein. This is a significant problem when dealing with multimeric proteins like the AQPs as presence of a single GFP-His_10_ tagged monomer causes the entire tetramer to bind the Ni-affinity column thus preventing further purification by reverse IMAC. As an alternative we therefore used the information gained from the GFP tagged AQPs to express and purify His-tagged AQP10 on a larger scale after growth in a computer controlled 15 liter bioreactor. To address the accumulation kinetics of AQP10-His_8_ in fermenter grown yeast cells we assessed the membranes isolated at different time points after induction from the bioreactor using western blotting and Coomassie staining after SDS-PAGE (Fig. 11a). In accordance with the shake flask experiments on the GFP-tagged versions (Fig. 2) the density of AQP10-His_8_ peaked at 48 hours after induction and remained at a high but reduced level until 95 hours after induction. This indicates that it is relevant to use GFP tagged membrane proteins to optimize production of the corresponding histidine tagged version. The solubilized AQP10-His_8_ fusion could be purified to near homogeneity by IMAC (Fig. 11b), and resulted in a monodisperse elution profile after size exclusion chromatography (Fig. 11c) at a yield of 26.4 mg AQP10 per 50 g yeast cells (weight weight). The purity of the final product was very high as determined by SDS-PAGE (Fig. 11d). It is also promising that the low amount of detergent required for AQP10-His_8_ purification is not in general inhibitory for structural studies.

The purified AQP proteins are only useful for biochemical and biophysical studies if they have preserved their ability to mediate transmembrane flow of water and possibly glycerol. It was therefore very encouraging that all purified AQPs after reconstitution into polymersomes were able to mediate an osmotically driven water flow and that only the aquaglyceroporins and to some extent the super-AQPs were able to confer a transmembrane flux of glycerol. All AQPs purified from our production platform were functional and showed the expected activities in polymersomes. These data also show that the GFP tag does not prevent the activity of the purified AQPs. Because we used the same amount of purified AQP-TEV-GFP-His_10_ protein for all transport assays we can assume that the transport activities reflect the permeability of each AQP. The obtained *P*_*f*_ values allows us to compare the relative water permeability of the different AQPs although comparison to *P*_*f*_ values reported in literature is not straight forward as these values are dependent on reconstitution system and conditions^54^.

To our knowledge this is the first time AQP activities have been compared for protein produced in the same expression platform and in the same reconstitution and stopped-flow setup. The permeabilities of the super-AQPs have for some time been questioned in the literature^9,27^ but we demonstrate that they both mediate osmotic water flow comparable to AQP3 and 6, respectively and also a transmembrane glycerol flux. This is in accordance with previous results showing glycerol permeation through AQP11^58^.

Apart from delivering AQP proteins for the present and future biophysical characterization, the relatively high number of AQPs exploited visualizes how general our expression platform is for production of active, high quality human membrane proteins. All-in-all the present paper demonstrates that our platform in a cheap and efficient manner is convenient for producing complex human membrane proteins like the AQPs in quantities and qualities requested for biophysical characterization such as crystallography.

## Methods

### Yeast strains

Production of recombinant AQPs was carried out in *S. cerevisiae* strain PAP1500 (α *ura3-52trp1::GAL10-GAL4 lys2-801 leu2Δ1 his3Δ200 pep4::HIS3prb1Δ1.6 R can1 GAL*)^30^.

### Plasmid constructions

Codon optimized human AQP cDNAs for *S. cerevisiae* were purchased from Genscript, USA. To C-terminally tag each AQP with a Tobacco Etch Virus cleavage site and a yEGFP-His_10_ sequence, we PCR amplified each codon optimized AQP cDNA with the following primers.

**AQP2fv**:5′ *TAAGATAATT* **ATGTGGGAATTGAGATCCATA** 3′

**AQP2rv**: 5′ **AGCCTTGGTACCTCTAGGTA** 3′

**AQP3fv**: *CTAAGATAATT* **ATGGGTAGACAAAAAGAATTAGT**

**AQP3rv**:5′ **AATTTGTTCCTTGTGTTTTACAT** 3′

**AQP6fw**: 5′ *CTAAGATAATT* **ATGGATGCTGTTGAACCAGGT** 3′

**AQP6rv**: 5′ **TACTGATTCCATTTCGACAGC** 3′

**AQP7fw**:5′ *CTAAGATAATT* **ATGGTCCAAGCCTCCGGTC** 3′

**AQP7rv**: 5′ **AAATTTTCAAAATGTTCCAAGGCCATAGATTC** 3′

**AQP8fw**: 5′ *CTAAGATAATT* **ATGTGTGAACCAGAATTTGGTAAT** 3′

**AQP8rv**: 5′ **TCTAGCCTTCAAGATTAATCTGG**3′

**AQP9fw**:5′ *CTAAGATAATT* **ATGCAACCAGAAGGTGCCG** 3′

**AQP9rv**:5′ **CATAATTACTGACAATTCGTACTTT**3′

**AQP10fw**:5′ *CTAAGATAATT* **ATGGTTTTTACACAAGCACCTG** 3′

**AQP10rv**:5′ **TAACTTGCATTCCAACATTTGT**3′

**AQP11fw**:5′ *CTAAGATAATT* **ATGTCCCCTTTGTTGGGTTTG** 3′

**AQP11rv**:5′ **TTCCTTCTTGTTGATAGTGTGG**3′

**AQP12fw**:5′ *CTAAGATAATT* **ATGGCAGGTTTGAACGTCAG**3′

**AQP12rv**:5′ **TGAAGAATGAGGACCTTCTAC**3′

Bold sequences are the template specific part of the primers, while the sequence in italics is the Kozak sequence from the yeast *PMR1* gene. The blue sequences are used for *in vivo* homologous recombination with the expression plasmid and the orange sequence is for recombination with the yeGFP3 PCR fragment to generate expression plasmids directly in the *S. cerevisiae* expression strain PAP1500.

The yeGFP3^59^ PCR fragments used for making the AQP-TEV-GFP-His_10_ fusions were generated with the following primers.

GFPupTEV:

5′ **ATGTCTAAAGGTGAAGAATTATTCACT**3′

GFPrecdo:

5′     **TTTGTACAATTCATCCATACCA**3′

The template specific sequences are shown in bold. The translational termination codon is in red, the ten histidine codons are in green, the sequence required for recombination with the AQP PCR products is shown in orange, while sequences required for recombination with the expression vector are shown in blue.

All PCR reactions were performed with AccuPol DNA polymerase (Amplicon, Denmark). Each AQP expression plasmid was generated by *in vivo* homologous recombination by transforming PAP1500 with an AQP PCR product plus a yeGFP3 PCR product and *Sal*I, *Hin*dIII and *Bam*HI digested pEMBLyex4^60^ expression vector, using the transformation protocol as described^61^. The correct nucleotide sequences of all expression constructs were verified by DNA sequencing at MWG Biotech, Germany.

### Small scale production of AQPs

Yeast cells were grown at 30 °C until saturation in 5 ml SD medium including leucine and lysine. 200 µl yeast culture was subsequently used to inoculate 5 ml SD medium supplemented with lysine at 30 °C until saturation. 50 ml of the same medium was inoculated with 5 ml pre-culture and growth continued until saturation. The 50 ml culture was used to inoculate 2 L expression medium (YP medium with 0.5% glucose and 3% glycerol) to OD_450_ = 0.05. Cells were incubated at room temperature until OD_450_ = 1.0 and subsequently transferred to 15 °C. AQP production was initiated after 15 minutes by supplementation with 200 ml 20% galactose dissolved in YP medium without glucose but with 3% glycerol.

### Temperature optimization of AQP production

Yeast cells were grown at room temperature as described above in 2 L of expression medium. At OD_450_ = 1.0, half of the culture was transferred to 15 °C and the other half to 30 °C. After thermo-equilibration, AQP production was induced by adding 220 ml of induction medium (20% galactose dissolved in YP medium containing 3% glycerol and no glucose) to each flask. Samples were collected 0, 4, 28, 52, 76 and 100 hours post induction. Crude membranes were isolated from cells harvested at each time point and analyzed by measuring GFP fluorescence in 25 µg crude membranes in a spectrofluorometer (Fluoroskan Ascent, Thermo Scientific) using buffer as a blank. Excitation was at 485 nm and emission at 520 nm. Fluorescence was converted to pmol AQP-GFP from a standard curve generated from purified GFP mixed with yeast membranes as previously established^31,32^.

### Production of Histidine tagged AQP10 in the Bioreactor

A single colony of transformed yeast cells was selectively propagated until saturation in 5 ml of glucose minimal medium supplemented with leucine and lysine. 200 µl of this culture was subsequently propagated in 5 ml of glucose minimal medium supplemented with lysine. The 5 ml were transferred to 50 ml of the same medium. Next day 1 l of glucose minimal medium supplemented with lysine was inoculated with the 50 ml pre-culture. After overnight growth the 1 l overnight culture was transferred to 10 liters of amino acid supplemented minimal medium with 3% glucose and 3% glycerol as carbon source and propagated in a 15 liters Applikon® bioreactor equipped with an ADI 1030 Bio Controller connected to a PC running the BioExpert® software (Applikon, Holland). The initial part of the fermentation was performed at 20 °C. The bioreactor was fed with glucose to a final concentration of 2% when the first amount of glucose had been metabolized. The pH of the growth medium was kept at 6.0 by computer-controlled addition of 1 M NH_4_OH. The shift from growth on glucose to growth on glycerol was monitored as a decrease in the rate of NH_4_OH consumption. At this point the bioreactor was cooled to 15 °C before induction of recombinant AQP production with 1 liter of 20% galactose dissolved in the initial growth medium lacking glucose. For time course expression experiments, 10 ml samples were taken out from the bioreactor at various time points after galactose induction and crude membranes were prepared as described below. Yeast cells were harvested after 72 h.

### Small scale purification of AQP-TEV-GFP-His_10_ fusion proteins

Yeast cells from 2 L cultures were harvested at 1,600 g for 10 minutes at 4 °C. Cells were lysed by glass bead homogenization in ice cold Lysis buffer (25 mM Imidazole 1 mM EDTA, 1 mM EGTA, 10% sucrose) containing 1 mM PMSF and 1 µg/ml of leupeptine, pepstatin and chymostatin as described previously^30^. The cell lysate was centrifuged at 4 °C at 3,000 g for 10 minutes and the supernatant exposed to ultracentrifugation at 40,000 rpm in a 70Ti rotor for 1.5 hours. Crude membrane pellets was re-suspended in lysis buffer containing protease inhibitors and kept at −80 °C until use. Crude membranes were solubilized for four hours at 4 °C by slow rotation in the detergent found to be most optimal from the detergent screen. The solubilized protein was isolated by ultracentrifugation at 4 °C for 30 minutes at 40,000 rpm in the 70Ti  rotor. The solubilized protein was diluted to a detergent concentration corresponding to 1.5 times CMC of the detergent used and incubated overnight in a beaker with Ni-resin (Genscript, USA). The beaker content was transferred to a 2 ml CellThru disposable column (Clontech, USA). After collection of the run through, the column was washed with Buffer containing 10 mM, 30 mM, 100 mM, 250 mM or 500 mM imidazole. All buffers contained detergent at a concentration corresponding to 1.5 times CMC. Fluorescence in each fraction was quantified using a spectrofluorometer (Fluoroskan Ascent, Thermo Scientific) using buffer as a blank. Excitation was at 485 nm and emission at 520 nm.

### Large-scale purification of Histidine tagged AQP10

For large-scale protein purification crude yeast membranes were isolated as for small-scale cultures, re-suspended in ice-cold solubilization buffer (20 mM Tris pH 7.5, 200 mM NaCl, 20% glycerol, 5 mM β-ME, 1 mM PMSF) supplemented with SIGMA*FAST* protease inhibitor cocktail (Sigma, USA) and solubilized for 4 h in the presence of 2% (w/v) n-decyl-β-D-maltopyranoside (DM; Anatrace). Solubilised material was clarified by 1-h centrifugation at 120,000 × *g*, diluted 2 × in immobilized metal affinity chromatography (IMAC) buffer (20 mM Tris pH 7.5, 200 mM NaCl, 20% glycerol, 5 mM β-ME and 0.2 (w/v)% DM) supplemented with 50 mM imidazole and filtered through a 0.45 μm filter. The sample was subsequently loaded onto a 5 mL Ni-NTA HisTrap HP column (GE Healthcare, USA), washed intensively in elution buffer (20 mM Tris pH 7.5, 200 mM NaCl, 20% glycerol, 5 mM β-ME and 1 (w/v)% n-octyl-β-D-glucopyranoside (bOG; Anatrace)) and eluted using a linear imidazole gradient (50–500 mM). Protein yields were quantified by ultraviolet–visible spectroscopy at A280 (ɛAQP10_His10_ = 40910 M^−1^ cm^−1^). Top fractions were pooled, concentrated to 1 mL using 100 kDa cut-off centrifugal concentrator (Sartorius) and loaded onto a size exclusion chromatography (SEC) Superdex increase 200 10/300 GL column (GE Healthcare) equilibrated in SEC buffer (20 mM Tris pH 7.5, 100 mM NaCl, 10% glycerol, 2 mM β-ME and 1% bOG). Purity of all samples collected during protein purification was analysed using SDS–PAGE.

### Live cell bioimaging

Localization of heterologously expressed GFP-tagged AQPs was determined by visualizing GFP fluorescence in whole cells at 1,000 × magnification, using a Nikon Eclipse E600 microscope coupled to an Optronics Magnafire model S99802 camera.

### Removal of N-glycosylation

Crude membranes containing 200 fluorescence units were incubated with 500 units of Endo-H (New Biolabs, USA) at 4 °C in Lysis buffer overnight alongside a negative control lacking addition of Endo-H. Samples were separated in 12% SDS-PAGE gels and visualized by in-gel fluorescence using a LAS 4000 Imager (GE Healthcare, USA).

### Detergent screens

Crude membranes were incubated in buffer B (25 mM Tris-HCl, 10 mM Imidazole, 0.5 M NaCl, 10% glycerol, pH 7.6) supplemented with protease inhibitors (1 mM PMSF and 1 *μ*g/ml Leupeptin, Chymostatin and Pepstatin, respectively) at protein:detergent:CHS ratios (w/w) of 1:1:0.3; 1:2:0.6 or 1:3:1.0. The screen included the following detergents FC-12, n-dodecylphosphocholine; FC-13, n-Tridecylphosphocholine; FC-14, n-Tetradecylphosphocholine; FC-16, n-Hexadecylphosphocholine; LDAO, Lauryldimethylamine N-oxide; Cymal-5, 5-cyclohexyl-1-pentyl-β-D-maltoside; DDM, n-dodecyl-β-D-maltopyranoside;DM, n-decyl-β-Dmaltopyranoside; Detergents were either of Anagrade quality and purchased from Affymetrix, UK or from Glycon, Germany. Solubilization was performed at slow rotation at 4 °C for 1 hour. Solubilized AQP-TEV-GFP-His_10_ protein was separated from un-solubilized cell debris by ultra-centrifugation at 70,000 rpm for 30 minutes at 4 °C in a Beckman Optima™TLX ultracentrifuge fitted with an S.N. 96U 826 rotor. Fluorescence was detected in microplates in a spectrofluorometer (Fluoroskan Ascent, Thermo Scientific) using buffer as a blank. Excitation was at 485 nm and emission at 520 nm. Solubilization efficiency was estimated as fluorescence in the supernatant divided by fluorescence in the crude membranes used for solubilization.

### Fluorescence-detection size exclusion chromatography

Solubilized crude membranes were analyzed by fluorescence-detection size exclusion chromatography (FSEC) on a Superose 6 Increase 200 10/300 GL column attached to an ÄKTA Purifier (GE Healthcare, USA), using FSEC buffer (20 mM TRIS-HCl, 0.15 M NaCl, 0.03% DDM). The effluent from the column was coupled to a fluorescence detector (Shimadzu Prominence RF-20A) to measure fluorescence and visualize the elution profile of the GFP tagged AQPs. To estimate the molecular weight of the solubilized AQP-TEV-GFPHis_10_ proteins, we used the HMW calibration kit from GE Healthcare dissolved at 20 mg/ml in FSEC buffer. The molecular masses were: Ovalbumin 43 kDa; Conalbumin 75 kDa; Aldolase 158 kDa; Ferritin 440 kDa; Thyroglobulin 669 kDA; Blue Dextran 2000 kDa. The elution volume for Blue Dextran defined the void volume.

### Ni-affinity purification

For purification the AQP-TEV-GFP-His_10_ fusions were solubilized in buffer B at slow rotation at 4 °C for 1 hour using the protein:detergent:CHS ratio (w/w/w) that gave the most monodisperse FSEC profile. Non-solubilized material was pelleted at 70,000 rpm in the Beckmann Optima TL200 ultracentrifuge for 30 minutes at 4 °C. Solubilized membranes were diluted in buffer B with protease inhibitors to a detergent concentration corresponding to 1.5 times CMC and incubated overnight in a glass beaker with 1 ml of Ni-NTA Agarose (Genscript, Germany) at 4 °C with slow magnetic stirring. The Agarose slurry was subsequently loaded onto a 2 ml CellThru disposable column (Clontech, USA). After collection of the run through, the column was washed with Buffer B containing 10 mM, 30 mM, 100 mM, 250 mM or 500 mM imidazole. All buffers contained detergent corresponding to 1.5 times CMC.

Fluorescence in each fraction was quantified using a spectrofluorometer (Fluoroskan Ascent, Thermo Scientific) using buffer as a blank. Excitation was at 485 nm and emission at 520 nm.

### TEV cleavage

Purified AQP-TEV-GFP-His_10_ fusion proteins were digested O/N in snakeskin dialysis bags (Thermo Scientific, USA) with dialysis buffer (20 mM phosphate pH 7.0, 200 mM NaCl, 1.5 x CMC detergent) and TEV protease^33^ at room temperature with a protein to TEV ratio of 1:10 (w/w). Digestion efficiency was estimated by in-gel fluorescence.

### Protein quantification

Protein concentrations in crude membranes were determined by the BCA assay^62^ according to the Manufacturer’s specifications (Sigma, USA) using chicken ovalbumin as a standard.

### SDS-PAGE and western blotting

SDS-PAGE separation of proteins, in-gel fluorescence and western blotting were done as previously described^31–33^. A mixture of two polyclonal anti-GFP-antibodies custom generated at Pineda, Germany, was used for AQP-TEV-GFP-His_10_ fusion proteins detection in western blots in conjunction with a horse radish conjugated anti-rabbit secondary antibody. Horse radish peroxidase conjugated Concanavalin-A (SigmaAldrich L6397) was used to identify glycosylated proteins after western blotting. Chemiluminescence detection was performed using the Immobilon Western Chemiluminescent HRP Substrate from Millipore® and the LAS4000 imager (GE Healthcare, USA).

### Proteopolymersome preparation and characterization

Poly(2-methyloxazoline)-block-poly(dimethylsiloxane) diblock co-polymer PDMS_34_PMOXA_11_ (PDMS-PMOXA) was purchased from DSM (Het Overloon 1, 6411 TE Heerlen, the Netherlands) and was used as received.

PDMS_34_PMOXA_11_ polymersomes and proteopolymersomes were prepared by the co-solvent method as described^63^. In short, 15 mg PDMS-PMOXA copolymer were dissolved in 50 µl ethanol, which was then added dropwise to 4450 µl Phosphate buffered saline (PBS, 10 mM, pH 7.2), followed by dialysis against PBS buffer for 24 hours with three buffer exchanges. For proteopolymersomes each AQP was incorporated by addition of detergent solubilized AQPs with a concentration of 25 µg before dialysis. Following dialysis all solutions were extruded 15 times through a 200 nm polycarbonate filter using an Avestin Extruder.

The size of extruded polymersomes and proteopolymersomes (hydrodynamic diameter) were determined by dynamic light scattering using ZetaSizer NanoZs (Malvern) with measuring temperature of 20 °C.

The water permeability of reconstituted AQPs were measured using a Bio-Logic SFM 300 stopped-flow device (Bio-Logic Science Instruments, France), with a monochromator at 517 nm and a cut off filter at 530 nm. For each stopped-flow test, 0.13 ml polymersome solution or proteopolymersome solution was mixed rapidly with 0.13 ml NaCl solution (0.5 M). Resulting proteopolymersome volume change rate measured by light scattering was recorded as a function of time with a deadtime of 5 ms. Thus, the effective extravesicular concentration is 0.25 M plus the sodium present in the 10 mM PBS resulting in a final NaCl concentration of 0.387 M. NaCl as an osmolyte provides a good signal to noise ratio and previous studies have shown that the optimal concentration of osmolyte is 0.3–0.6 M NaCl^44^. This condition allows for fitting the stopped flow signal to more than 95% accuracy^44^.

Proteopolymersome volume reduction was due to water transport outward as an effect of the change in osmolarity and the water permeability of the proteopolymersome. The results are reported as an average of 10 measurements. The average rate constant k_i_ of the stopped flow signal was determined by fitting of the normalized light scattering curves to a double order exponential function where the initial rate constant is directly proportional to the water flux though the proteopolymersome.

In order to test the glycerol flux through AQP channels, 3 ml extruded polymersomes (polymeric vesicles) were incubated with 3 ml 2 M glycerol overnight at 4 °C to equilibrate the glycerol concentration across the polymeric vesicles. After incubation, the dimensions of the polymeric vesicles were determined by dynamic light scattering. Polymeric vesicles only swell if the tested AQP can mediate a glycerol flux otherwise it will shrink due to the outward osmotic gradient. The water flux trough AQP channels after incubation was tested in a similar way using the stopped-flow method, by mixing with 0.5 M NaCl that exhibits the same osmotic pressure as 1 M glycerol concentration, incubating with the polymer vesicles. For AQPs that only transport water the k_i_ with or without prior glycerol incubation will be identical when exposed to NaCl, while the k_i_ for the Aquaglyceroporins will decrease after glycerol incubation. The Δ_ki_ (ki without glycerol equilibration– k_i_ after glycerol equilibration) will then be zero for an orthodox AQP but larger than one for an aquaglyceroporin.

The water flux trough AQP channels after incubation was tested in a similar way using the stopped-flow method, by mixing with 0.5 M NaCl that exhibits the same osmotic pressure as 1 M glycerol concentration^64–66^.

## Electronic supplementary material

Supplementary Figures
