# Supplementary material for: Purification and functional comparison of nine human Aquaporins produced in Saccharomyces cerevisiae for the purpose of biophysical characterization
Source: Sci Rep. 2017 Dec 4;7:16899. doi: 10.1038/s41598-017-17095-6 (PMC5715081; doi:10.1038/s41598-017-17095-6)
Supplement: Supplementary file 1 — Supplementary Figures [file 41598_2017_17095_MOESM1_ESM.pdf]

Supplementary Files

(Figures 1-6)

Purification and functional comparison of nine human Aquaporins produced in *Saccharomyces cerevisiae* for the purpose of biophysical characterization

Frederik Bühring Bjørkskov[1#], Simon Lyngaa Krabbe[1#], Casper Normann Nurup[1#], Julie Winkel Missel[2], Mariana Spulper[4], Julie Bomholt[3], Karen Molbaek[1], Claus Helix-Nielsen[4,5,6], Kamil Gotfryd[2], Pontus Gourdon[2,7] and Per Amstrup Pedersen[1*]

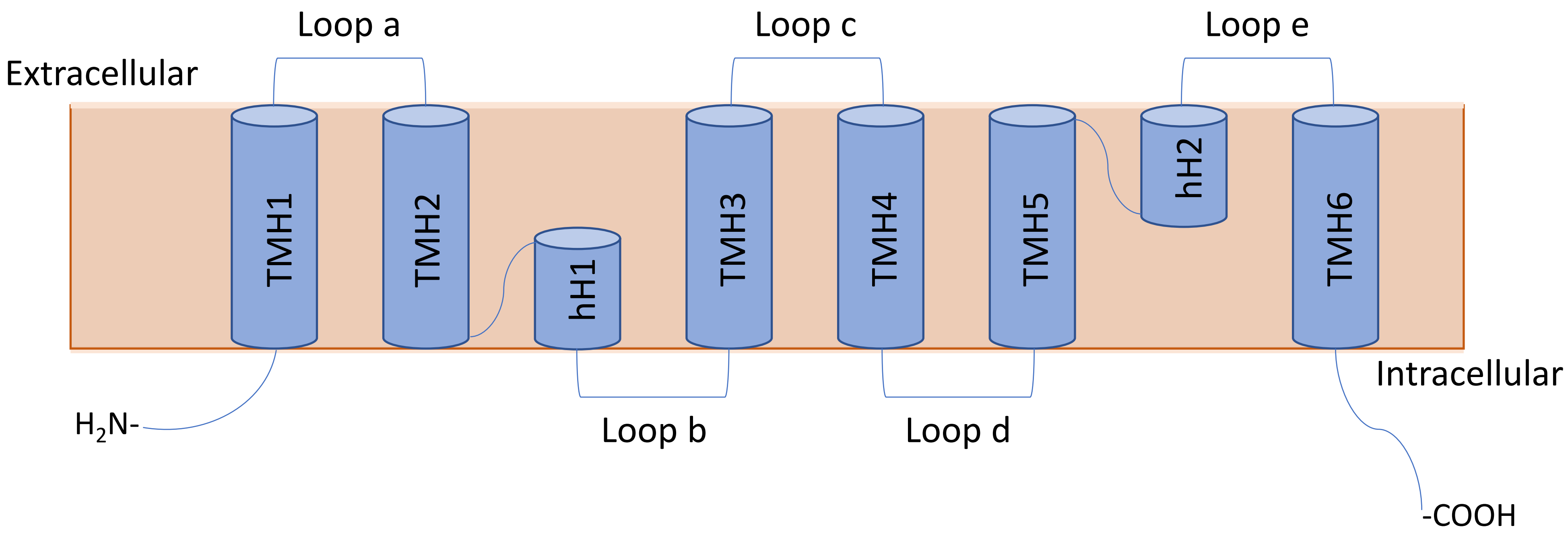

Supplementary Figure 1: Membrane topology of AQPs. Membrane topology of the AQP monomer showing the six transmembrane helixes TMH1-6, the two half-helixes hH1-2 carrying the NPA sequences conserved in most AQPs and the membrane connecting loops designated a, b, c, d and e.

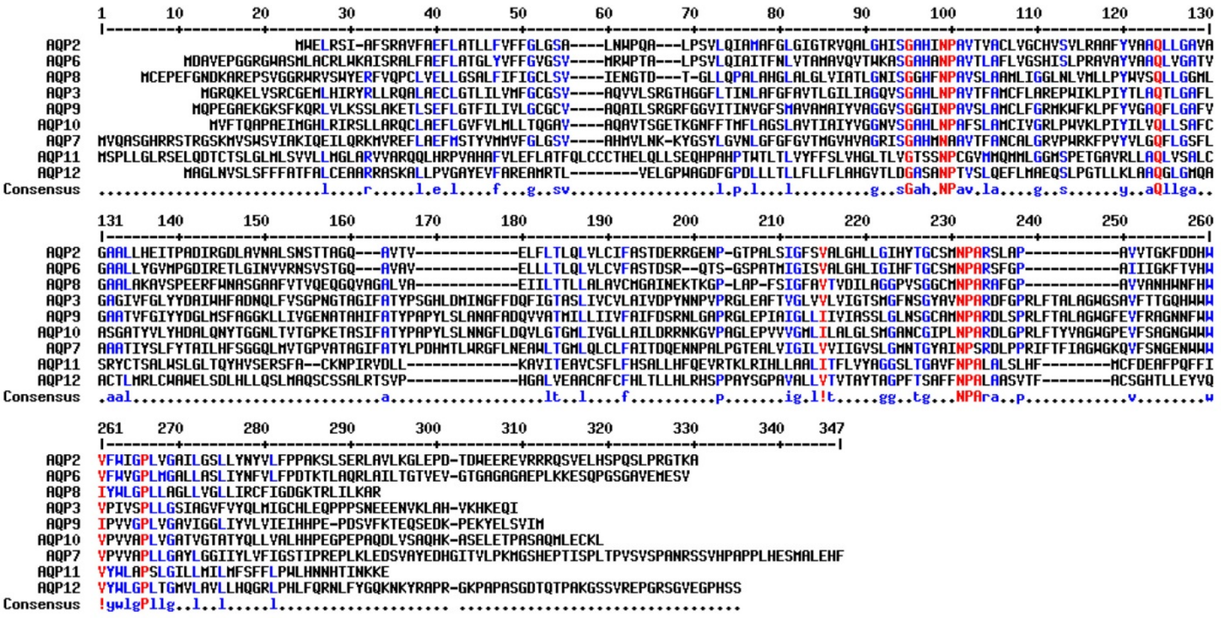

Supplementary Figure 2: Alignment of the nine produced AQPs. The primary structures of the produced AQPs were aligned using Multalin (multalin.toulouse.inra.fr/multalin/).Conserved amino acids are depicted in red while amino acids in blue represent conservation of physico-chemical properties.

Bioinformatics of human Aquaporins

AQP2

MWELRSIAFSRAVFAEFLATLLFVFFGLGSALNWPQALPSVLQIAMAFGLGIGTRVQALGHISG
AHINPAVTVACLVGCHVSVLRAAFYVAAQLLGAVAGAALLHEITPADIRGDLAVNALSNSTT
AGQAVTVELFLTLQLVLCIFASTDERRGENPGTPALSIGFSVALGHLLGIHYTGCSMNPARSLA
PAVVTGKFDDHWVFWIGPLVGAILGSLLYNYVLFPPAKSLSERLAVLKGLEPDTDWEEREVR
RRQSVELHSPQSLPRGTKA

AQP3

MGRQKELVSRCGEMLHIRYRLLRQALAECLGTLILVMFGCGSVAQVVLSRGTHGGFLTINLAF
GFAVTLGILIAGQVSGAHLNPAVTFAMCFLAREPWIKLPIYTLAQTLGAFLGAGIVFGLYYDAI
WHFADNQLFVSGPNGTAGIFATYPSGHLDMINGFFDQFIGTASLIVCVLAIVDPYNNPVPRGLE
AFTVGLVVLVIGTSMGFNSGYAVNPARDFGPRLFTALAGWGSAVFTTGQHWWWVPIVSPLL
GSIAGVFVYQLMIGCHLEQPPPSNEEENVKLAHVKHKEQI

AQP6

MDAVEPGGRGWASMLACRLWKAISRALFAEFLATGLYVFFGVGSVMRWPTALPSVLQIAITF
NLVTAMAVQVTWKASGAHANPAVTLAFLVGSHISLPRAVAYVAAQLVGATVGAALLYGVM
PGDIRETLGINVVRNSVSTGQAVAVELLLTLQLVLCVFASTDSRQTSGSPATMIGISVALGHLIG
IHFTGCSMNPARSFGPAIIIGKFTVHWVFWVGPLMGALLASLIYNFVLFPDTKTLAQRLAILTGT
VEVGTGAGAGAEPLKKESQPGSGAVEMESV

AQP7

MVQASGHRRSTRGSKMVSWSVIAKIQEILQRKMVREFLAEFMSTYVMMVFGLGSVAHMVLN
KKYGSYLGVNLGFGFGVTMGVHVAGRISGAHMNAAVTFANCALGRVPWRKFPVYVLGQFL
GSFLAAATIYSLFYTAILHFSGGQLMVTGPVATAGIFATYLPDHMTLWRGFLNEAWLTGMLQ
LCLFAITDQENNPALPGTEALVIGILVVIIGVSLGMNTGYAINPSRDLPPRIFTFIAGWGKQVFSN
GENWWWVPVVAPLLGAYLGGIIYLVFIGSTIPREPLKLEDSVAYEDHGITVLPKMGSHEPTISP
LTPVSVSPANRSSVHPAPPLHESMALEHF

AQP8

MCEPEFGNDKAREPSVGGRWRVSWYERFVQPCLVELLGSALFIFIGCLSVIENGTDTGLLQPAL
AHGLALGLVIATLGNISGGHFNPAVSLAAMLIGGLNLVMLLPYWVSQLLGGMLGAALAKAVS
PEERFWNASGAAFVTVQEQGQVAGALVAEIILTTLLALAVCMGAINEKTKGPLAPFSIGFAVT
VDILAGGPVSGGCMNPARAFGPAVVANHWNFHWIYWLGPLLAGLLVGLLIRCFIGDGKTRLI
LKAR

AQP9

MQPEGAEKGKSFKQRLVLKSSLAKETLSEFLGTFILIVLGCGCVAQAILSRGRFGGVITINVGFS
MAVAMAIYVAGGVSGGHINPAVSLAMCLFGRMKWFKLPFYVGAQFLGAFVGAATVFGIYYD
GLMSFAGGKLLIVGENATAHIFATYPAPYLSLANAFADQVVATMILLIIVFAIFDSRNLGAPRG
LEPIAIGLLIIVIASSLGLNSGCAMNPARDLSPRLFTALAGWGFEVFRAGNNFWWIPVVGPLVG
AVIGGLIYVLVIEIHHPEPDSVFKTEQSEDKPEKYELSVIM

AQP10

MVFTQAPAEIMGHLRIRSLLARQCLAEFLGVFVLMLLTQGAVAQAVTSGETKGNFFTMFLAG
SLAVTIAIYVGGNVSGAHLNPAFSLAMCIVGRLPWVKLPIYILVQLLSAFCASGATYVLYHDAL
QNYTGGNLTVTGPKETASIFATYPAPYLSLNNGFLDQVLGTGMLIVGLLAILDRRNKGVPAGL
EPVVVGMLILALGLSMGANCGIPLNPARDLGPRLFTYVAGWGPEVFSAGNGWWWVPVVAPL
VGATVGTATYQLLVALHHPEGPEPAQDLVSAQHKASELETPASAQMLECKL

AQP11

MSPLLGLRSELQDTCTSLGLMLSVVLLMGLARVVARQQLHRPVAHAFVLEFLATFQLCCCTH
ELQLLSEQHPAHPTWTLTLVYFFSLVHGLTLVGTSSNPCGVMMQMMLGGMSPETGAVRLLA
QLVSALCSRYCTSALWSLGLTQYHVSERSFACKNPIRVDLLKAVITEAVCSFLFHSALLHFQEV
RTKLRIHLLAALITFLVYAGGSLTGAVFNPALALSLHFMCFDEAFPQFFIVYWLAPSLGILLMIL
MFSFFLPWLHNNHTINKKE

AQP12

MAGLNVSLSFFFATFALCEAARRASKALLPVGAYEVFAREAMRTLVELGPWAGDFGPDLLLT
LLFLLFLAHGVTLDGASANPTVSLQEFLMAEQSLPGTLLKLAAQGLGMQAACTLMRLCWAW
ELSDLHLLQSLMAQSCSSALRTSVPHGALVEAACAFCFHLTLLHLRHSPPAYSGPAVALLVTV
TAYTAGPFTSAFFNPALAASVTFACSGHTLLEYVQVYWLGPLTGMVLAVLLHQGRLPHLFQR
NLFYGQKNKYRAPRGKPAPASGDTQTPAKGSSVREPGRSGVEGPHSS

Supplementary Figure 3: Prediction of N- and O-glycosylation sites in the purified AQPs. The primary structures of the nine AQPs were analyzed for location of transmembane segments and N- and O-glycosylation sites as described in Methods. Yellow, cytoplasmically located N- and C-terminals; Red, predicted transmembrane segments; N, a predicted N-glycosylation site located on the extra cellular side of the membrane; N, a predicted N-glycosylation site not located on the extra cellular side of the membrane; N, a predicted N-glycosylation carrying a proline in its consensus sequence; S, T, predicted O-glycosylation sites.

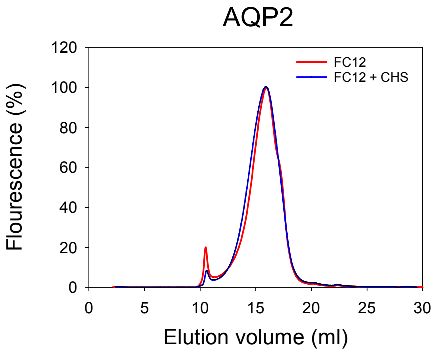
AQP2
FC12
FC12 + CHS
Flourescence (%)
Elution volume (ml)

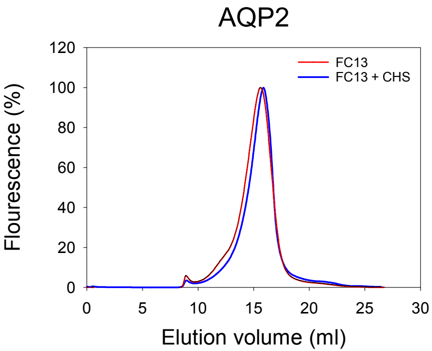
AQP2
FC13
FC13 + CHS
Flourescence (%)
Elution volume (ml)

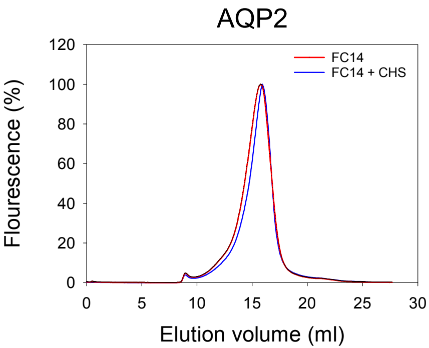
AQP2
FC14
FC14 + CHS
Flourescence (%)
Elution volume (ml)

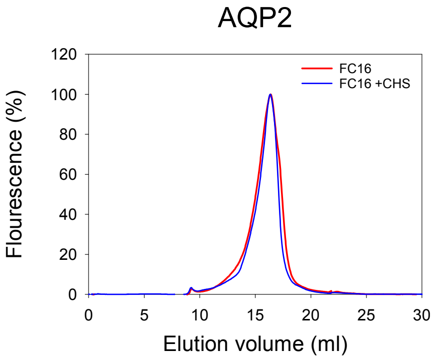
AQP2
FC16
FC16 +CHS
Flourescence (%)
Elution volume (ml)

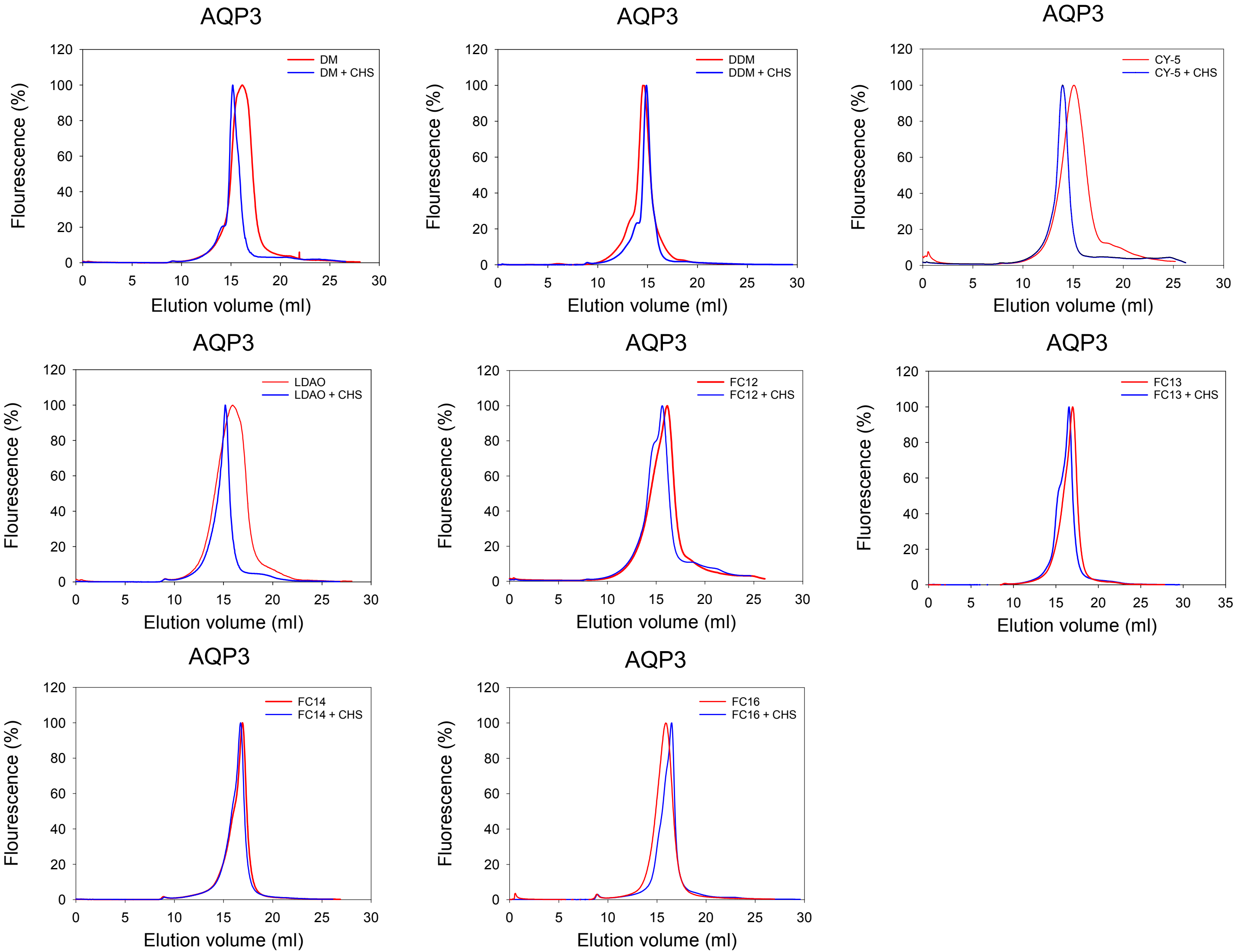

AQP3
DM
DM + CHS
Flourescence (%)
Elution volume (ml)
AQP3
DDM
DDM + CHS
Flourescence (%)
Elution volume (ml)
AQP3
CY-5
CY-5 + CHS
Flourescence (%)
Elution volume (ml)
AQP3
LDAO
LDAO + CHS
Flourescence (%)
Elution volume (ml)
AQP3
FC12
FC12 + CHS
Flourescence (%)
Elution volume (ml)
AQP3
FC13
FC13 + CHS
Fluorescence (%)
Elution volume (ml)
AQP3
FC14
FC14 + CHS
Flourescence (%)
Elution volume (ml)
AQP3
FC16
FC16 + CHS
Fluorescence (%)
Elution volume (ml)

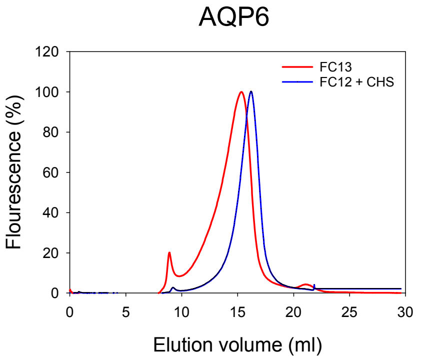
AQP6
Flourescence (%)
Elution volume (ml)
FC13
FC12 + CHS

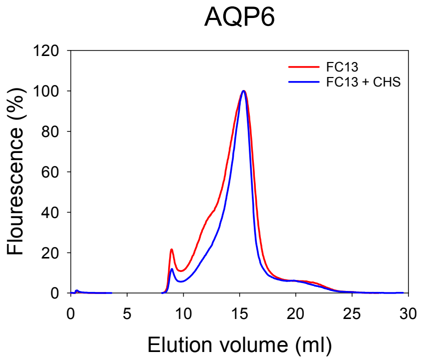
AQP6
Flourescence (%)
Elution volume (ml)
FC13
FC13 + CHS

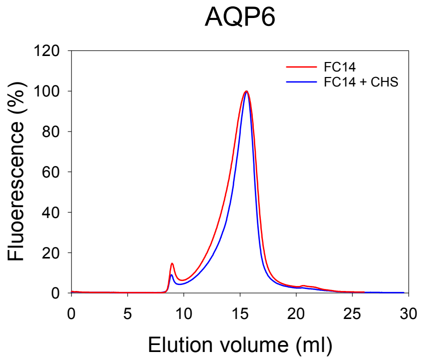
AQP6
Fluoerescence (%)
Elution volume (ml)
FC14
FC14 + CHS

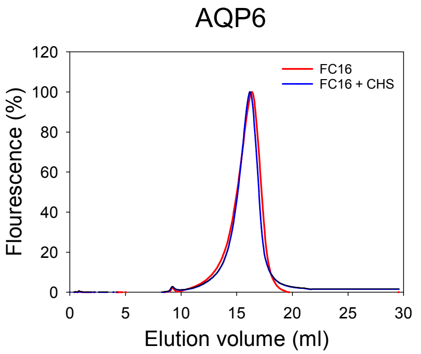
AQP6
Flourescence (%)
Elution volume (ml)
FC16
FC16 + CHS

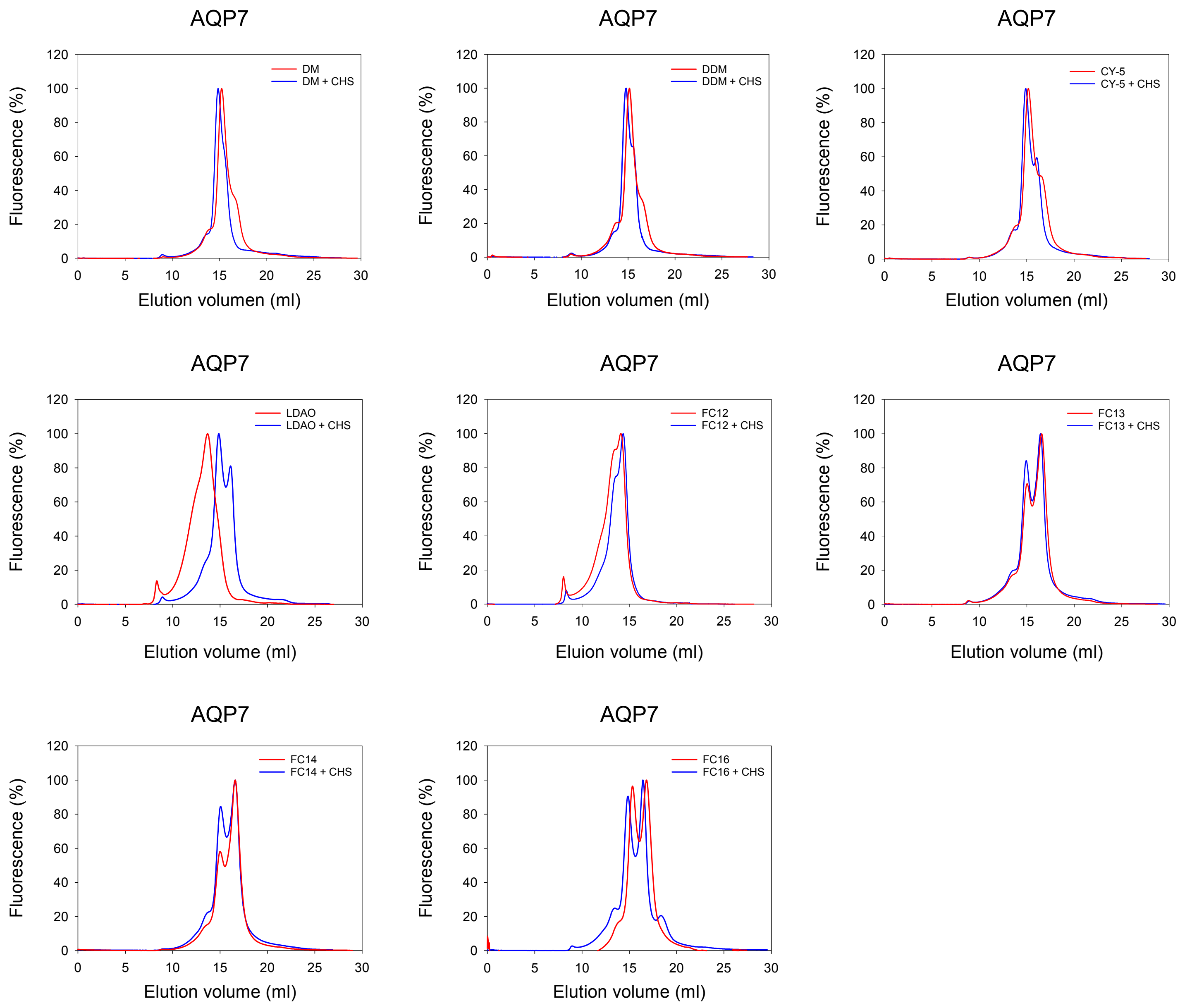
AQP7
DM
DM + CHS
AQP7
DDM
DDM + CHS
AQP7
CY-5
CY-5 + CHS
AQP7
LDAO
LDAO + CHS
AQP7
FC12
FC12 + CHS
AQP7
FC13
FC13 + CHS
AQP7
FC14
FC14 + CHS
AQP7
FC16
FC16 + CHS
Fluorescence (%)
Elution volumen (ml)
Elution volume (ml)
Eluion volume (ml)
0
20
40
60
80
100
120
0
5
10
15
20
25
30

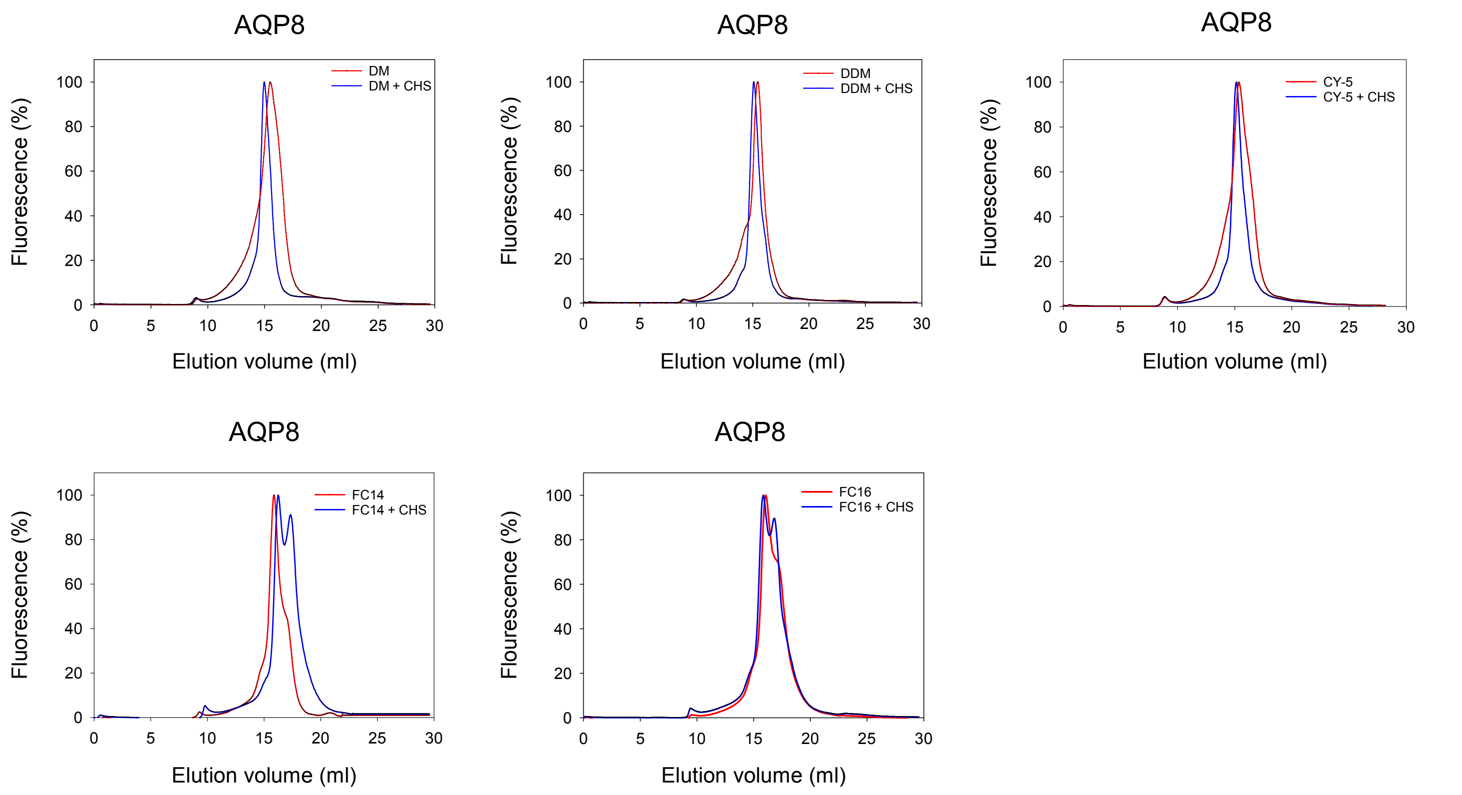
AQP8
DM
DM + CHS
Fluorescence (%)
Elution volume (ml)
AQP8
DDM
DDM + CHS
Fluorescence (%)
Elution volume (ml)
AQP8
CY-5
CY-5 + CHS
Fluorescence (%)
Elution volume (ml)
AQP8
FC14
FC14 + CHS
Fluorescence (%)
Elution volume (ml)
AQP8
FC16
FC16 + CHS
Flourescence (%)
Elution volume (ml)
0
20
40
60
80
100
0
5
10
15
20
25
30

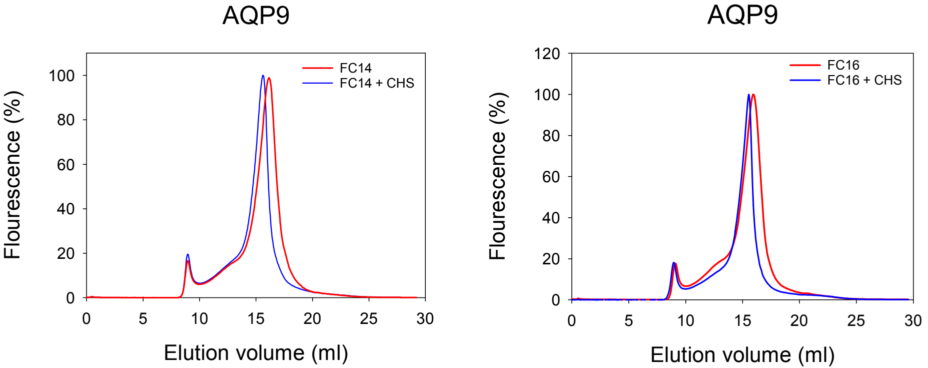

AQP9
FC14
FC14 + CHS
Flourescence (%)
Elution volume (ml)
0
20
40
60
80
100
0
5
10
15
20
25
30
AQP9
FC16
FC16 + CHS
Flourescence (%)
Elution volume (ml)
0
20
40
60
80
100
120
0
5
10
15
20
25
30

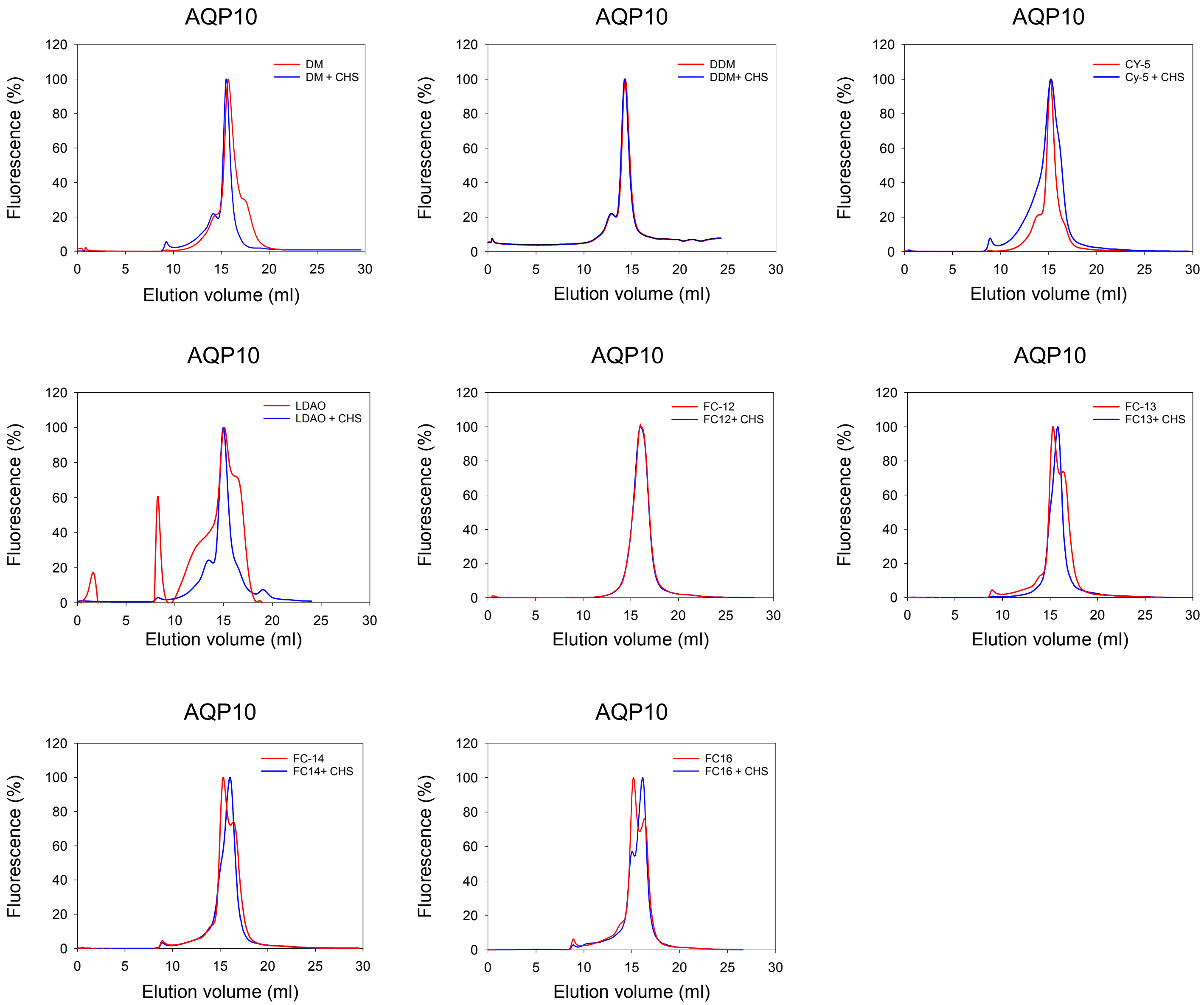
AQP10
DM
DM + CHS
AQP10
DDM
DDM+ CHS
AQP10
CY-5
Cy-5 + CHS
AQP10
LDAO
LDAO + CHS
AQP10
FC-12
FC12+ CHS
AQP10
FC-13
FC13+ CHS
AQP10
FC-14
FC14+ CHS
AQP10
FC16
FC16 + CHS
Fluorescence (%)
Elution volume (ml)

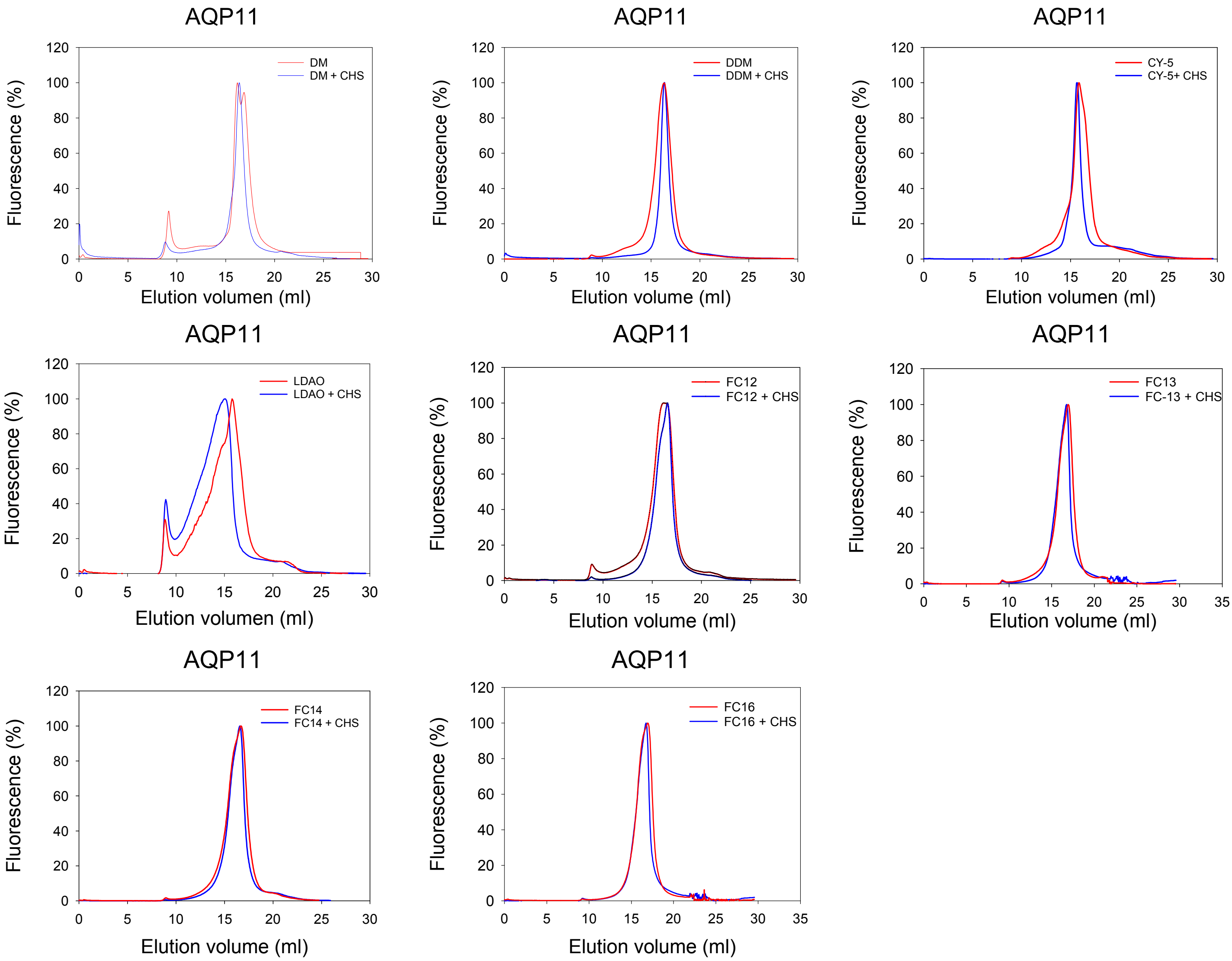
AQP11
DM
DM + CHS
Fluorescence (%)
Elution volumen (ml)
AQP11
DDM
DDM + CHS
Fluorescence (%)
Elution volume (ml)
AQP11
CY-5
CY-5+ CHS
Fluorescence (%)
Elution volumen (ml)
AQP11
LDAO
LDAO + CHS
Fluorescence (%)
Elution volumen (ml)
AQP11
FC12
FC12 + CHS
Fluorescence (%)
Elution volume (ml)
AQP11
FC13
FC-13 + CHS
Fluorescence (%)
Elution volume (ml)
AQP11
FC14
FC14 + CHS
Fluorescence (%)
Elution volume (ml)
AQP11
FC16
FC16 + CHS
Fluorescence (%)
Elution volume (ml)

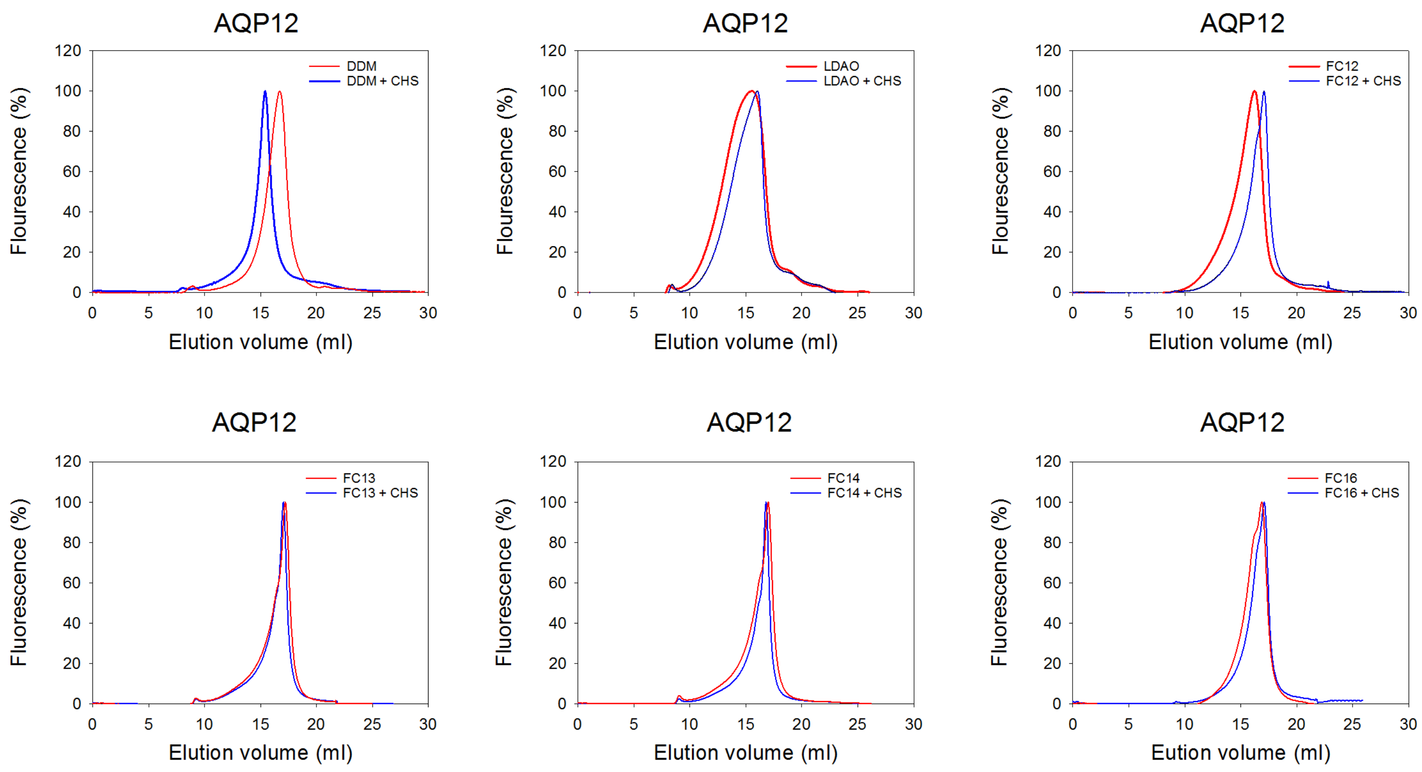

Supplementary Figure 4: Fluorescence-detection size exclusion chromatography of detergent solubilized human AQPs. Detergent solubilized membrane proteins were separated on a Superose 6 increase 200 10/300 GL column as described in Methods. The figures show the FSEC profiles for the detergents that were not used for the purification of the AQPs shown in Figure 9 and for the same detergents supplemented with Cholesteryl hemi-succinate (CHS). Fluorescence has been normalized to the peak value in each profile. The void volume is around 8 ml.

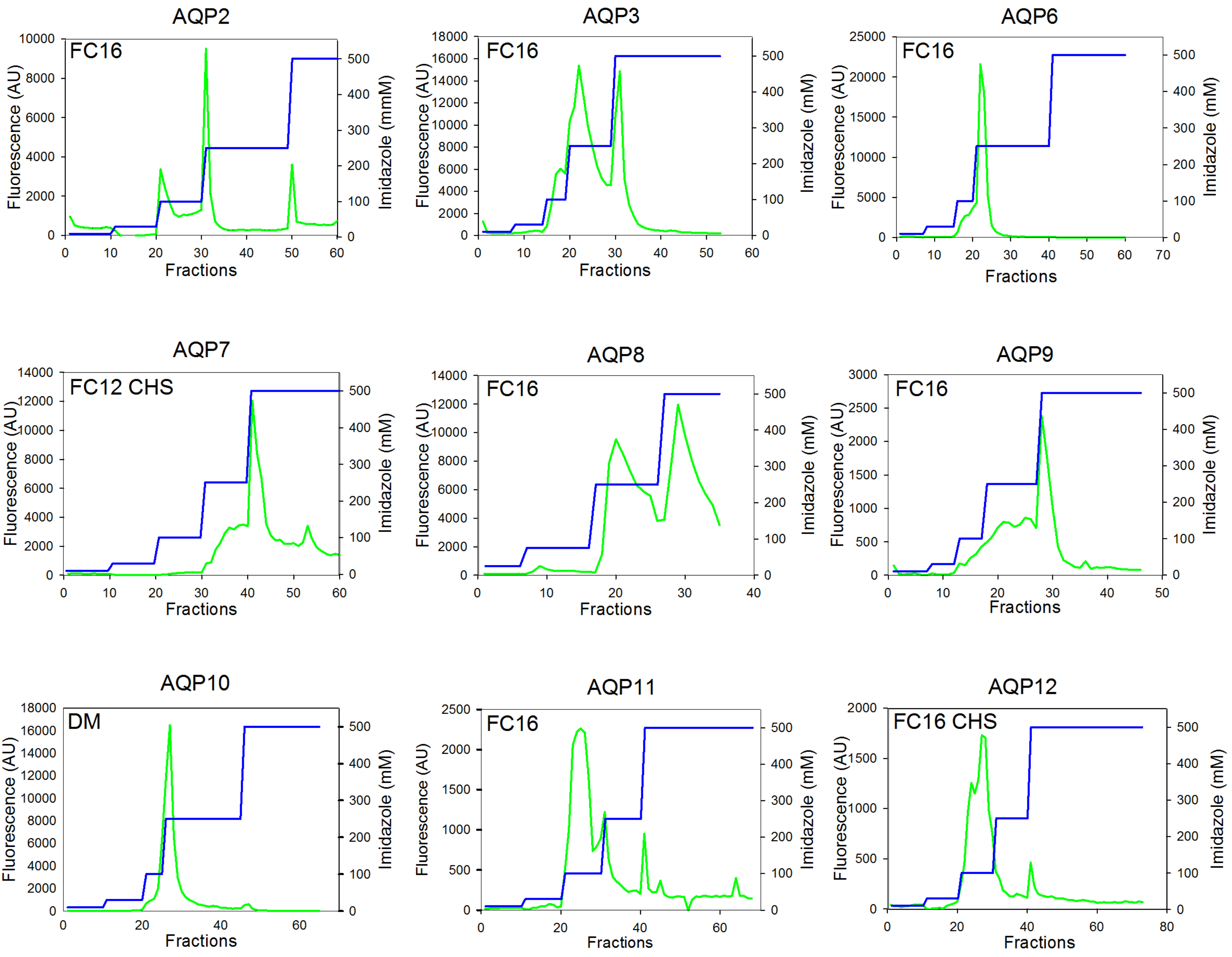

Supplementary Figure 5: Ni-affinity purification of detergent solubilized AQPs. Based on the data from Figure 7 the AQPs were solubilized in the detergent shown in each sub-figure and subsequently incubated with Ni-resin over night at 4°C as described in Methods. The Ni-resin was poured on a column and the AQPs were eluted using the indicated imidazole step gradient (blue). Fluorescence was measured in all fractions to determine the elution profile of each aquaporin (green).

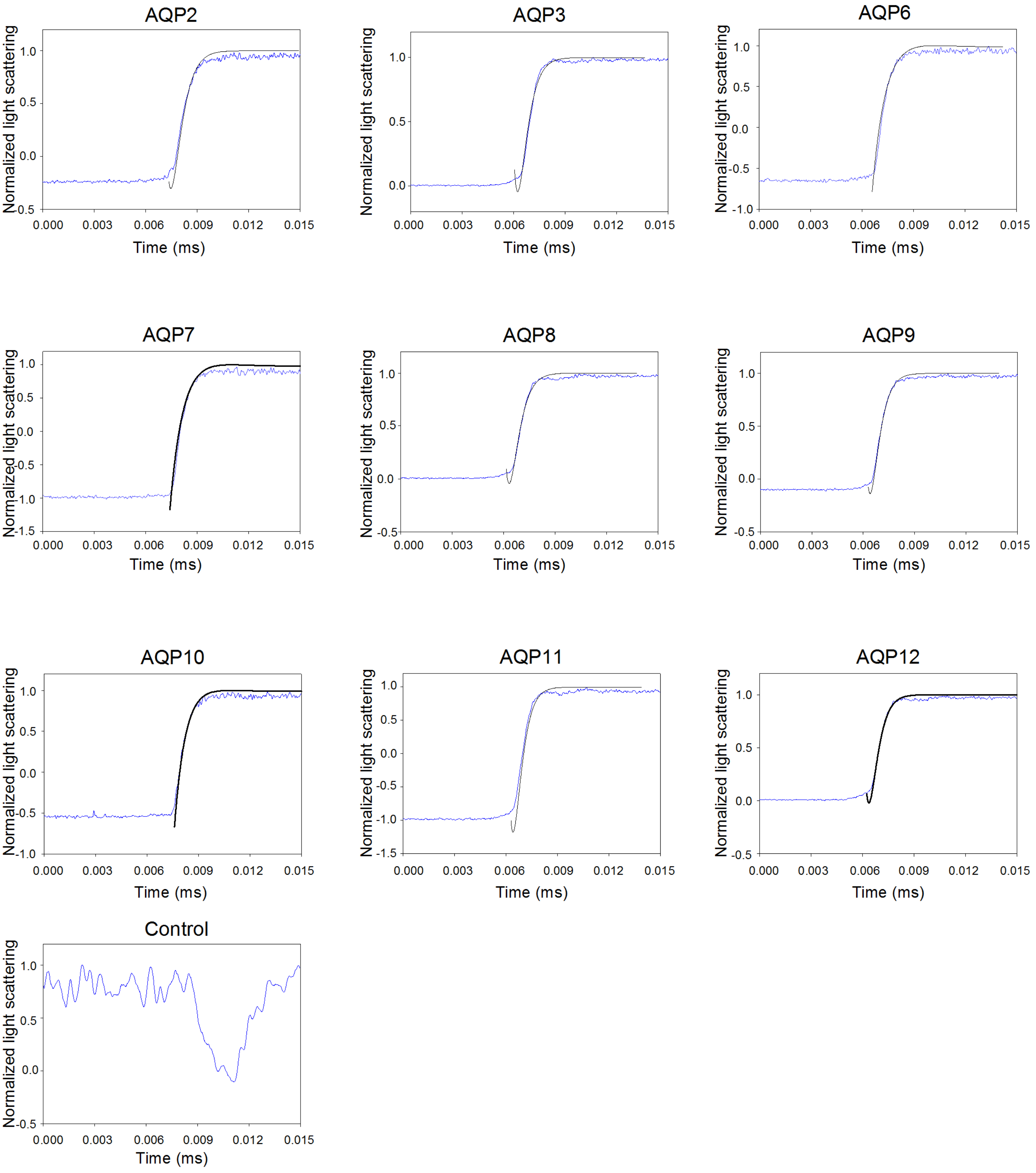

Supplementary Figure 6: Each human AQP mediates a water flux after reconstitution into proteopolymersomes. Each aquaporin was reconstituted into proteopolymersomes and exposed to a hyperosmolar shock as described in Methods. Polymersomes without any AQP were included as a negative control. Graphs in blue show the normalized light scattering of the proteopolymersomes after hyperosmolar shock. Graphs in black shows a fit to a second order exponential function used to determine the average rate constant k_i of the stopped-flow signal. The rate constant k_i is directly proportional to the water flux though the proteopolymersome. All AQPs are active and mediate the transport of water.

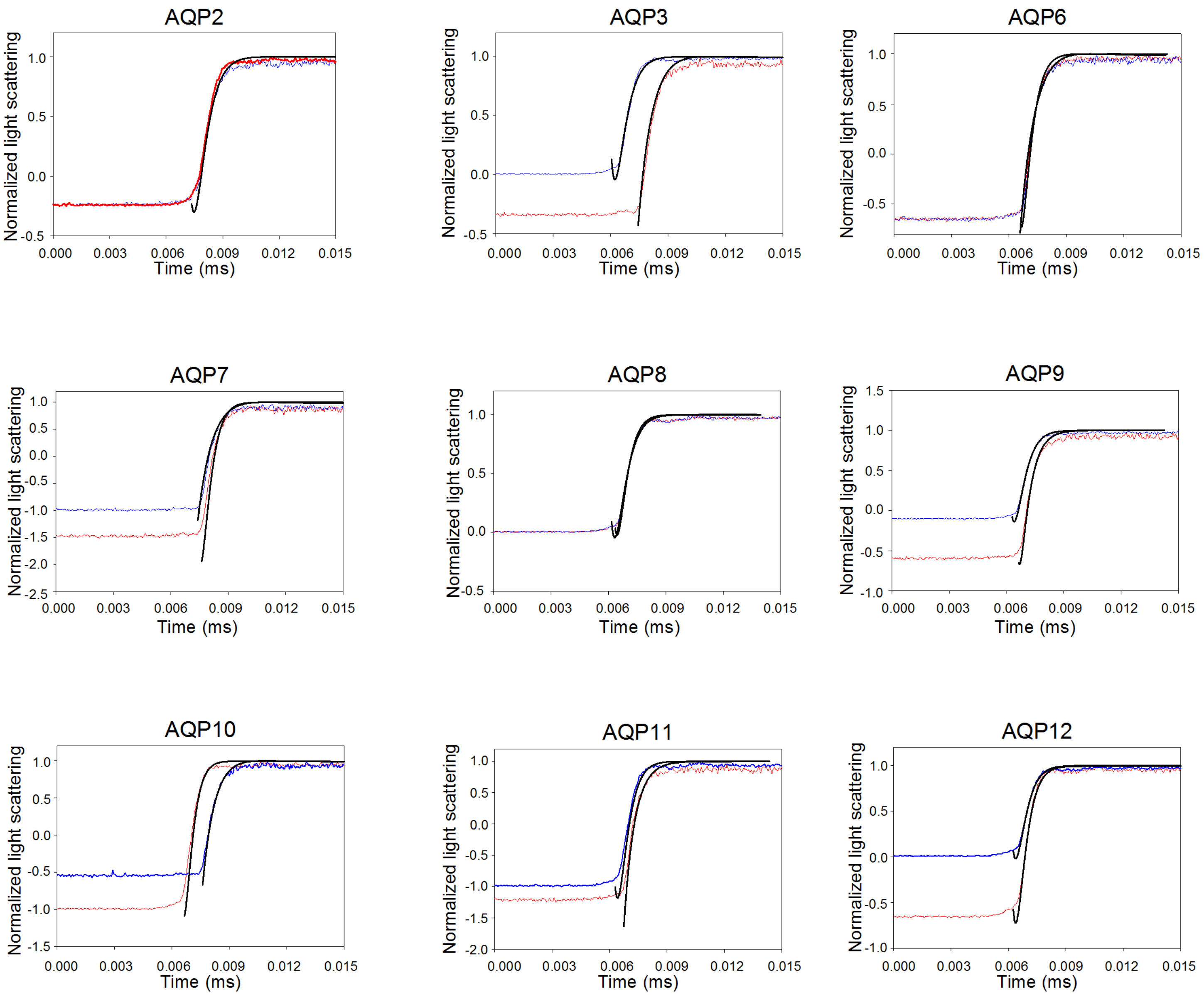

Supplementary Figure 7: Human AQPs 3, 7, 9, 10, 11 and 12 mediate transport of glycerol after reconstitution into proteopolymersomes. Comparison of water flux with (graphs in red) or without (graphs in blue) incubation with glycerol prior to hyperosmolar shock. Graphs in black shows the fit to a second order exponential function used to determine the k_i-value which is directly proportional to the water flux through the proteopolymersome. The slopes of the curves are identical for AQP2, 6 and 8, while there is a distinct difference in the slopes for AQP3, 7, 9, 10, 11, and 12, indicating that these AQPs have mediated uptake of glycerol into the proteopolymersome.
